# Culture media composition influences patient-derived organoid ability to predict therapeutic responses in gastrointestinal cancers

**DOI:** 10.1172/jci.insight.158060

**Published:** 2022-11-22

**Authors:** Tara L. Hogenson, Hao Xie, William J. Phillips, Merih D. Toruner, Jenny J. Li, Isaac P. Horn, Devin J. Kennedy, Luciana L. Almada, David L. Marks, Ryan M. Carr, Murat Toruner, Ashley N. Sigafoos, Amanda N. Koenig-Kappes, Rachel L.O. Olson, Ezequiel J. Tolosa, Cheng Zhang, Hu Li, Jason D. Doles, Jonathan Bleeker, Michael T. Barrett, James H. Boyum, Benjamin R. Kipp, Amit Mahipal, Joleen M. Hubbard, Temperance J. Scheffler Hanson, Gloria M. Petersen, Surendra Dasari, Ann L. Oberg, Mark J. Truty, Rondell P. Graham, Michael J. Levy, Mojun Zhu, Daniel D. Billadeau, Alex A. Adjei, Nelson Dusetti, Juan L. Iovanna, Tanios S. Bekaii-Saab, Wen Wee Ma, Martin E. Fernandez-Zapico

**Affiliations:** 1 Schulze Center for Novel Therapeutics, Division of Oncology Research, Mayo Clinic, Rochester, Minnesota, USA.; 2 Department of Gastrointestinal Oncology, H Lee Moffitt Cancer Center and Research Institute, Tampa, Florida, USA.; 3 Division of Medical Oncology, Department of Oncology,; 4 Department of Pharmacology, and; 5 Department of Biochemistry and Molecular Biology, Mayo Clinic, Rochester, Minnesota, USA.; 6 Sanford Research, Oncology, Sanford Health, Sioux Falls, South Dakota, USA.; 7 Division of Hematology/Oncology, Mayo Clinic, Phoenix, Arizona, USA.; 8 Department of Radiology,; 9 Department of Laboratory Medicine and Pathology,; 10 Division of Epidemiology, Department of Quantitative Health Sciences,; 11 Division of Computational Biology, Department of Quantitative Health Sciences, and; 12 Department of Surgery, Mayo Clinic, Rochester, Minnesota, USA.; 13 Division of Anatomic Pathology, Mayo Clinic, Rochester, Minnesota, USA.; 14 Division of Gastroenterology and Hepatology, Department of Medicine, Mayo Clinic, Rochester, Minnesota, USA.; 15 Cancer Research Center of Marseille (CRCM), INSERM U1068, CNRS UMR 7258, Aix-Marseille Université and Institut Paoli-Calmettes, Parc Scientifique et Technologique de Luminy, Marseille, France.

**Keywords:** Oncology, Therapeutics, Colorectal cancer, Drug therapy, Liver cancer

## Abstract

**BACKGROUND:**

A patient-derived organoid (PDO) platform may serve as a promising tool for translational cancer research. In this study, we evaluated PDO’s ability to predict clinical response to gastrointestinal (GI) cancers.

**METHODS:**

We generated PDOs from primary and metastatic lesions of patients with GI cancers, including pancreatic ductal adenocarcinoma, colorectal adenocarcinoma, and cholangiocarcinoma. We compared PDO response with the observed clinical response for donor patients to the same treatments.

**RESULTS:**

We report an approximately 80% concordance rate between PDO and donor tumor response. Importantly, we found a profound influence of culture media on PDO phenotype, where we showed a significant difference in response to standard-of-care chemotherapies, distinct morphologies, and transcriptomes between media within the same PDO cultures.

**CONCLUSION:**

While we demonstrate a high concordance rate between donor tumor and PDO, these studies also showed the important role of culture media when using PDOs to inform treatment selection and predict response across a spectrum of GI cancers.

**TRIAL REGISTRATION:**

Not applicable.

**FUNDING:**

The Joan F. & Richard A. Abdoo Family Fund in Colorectal Cancer Research, GI Cancer program of the Mayo Clinic Cancer Center, Mayo Clinic SPORE in Pancreatic Cancer, Center of Individualized Medicine (Mayo Clinic), Department of Laboratory Medicine and Pathology (Mayo Clinic), Incyte Pharmaceuticals and Mayo Clinic Hepatobiliary SPORE, University of Minnesota-Mayo Clinic Partnership, and the Early Therapeutic program (Department of Oncology, Mayo Clinic).

## Graphical abstract

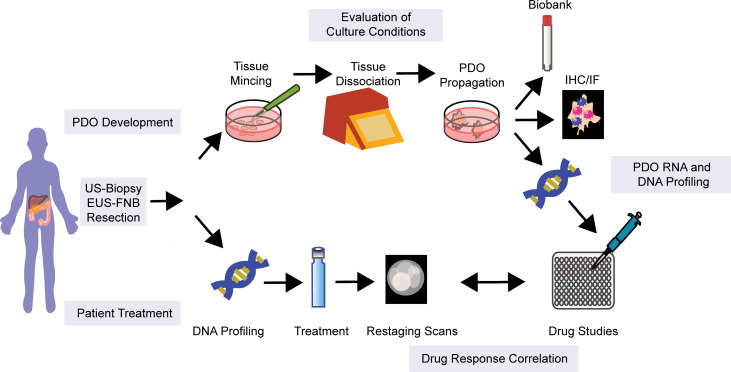

## Introduction

Gastrointestinal (GI) cancers, including gastric, hepatic, esophageal, pancreatic, and colorectal, account for one-fourth of the global cancer incidence and one-third of cancer-related deaths (1). Except for colorectal cancer, prognosis is often poor due to diagnosis at advanced stages (2). Nonsurgical treatments for advanced GI cancers include radiotherapy and chemotherapy (3). Genetic profiling of tumor tissue is commonly used to identify druggable mutations and to select patients for clinical trials. Genotype-matched clinical trials are associated with an increased tumor response rate; however, there is a need for new therapeutic options to improve prognosis (4). Currently, only 5% of patients who undergo tumor profiling are enrolled in a clinical trial (4, 5). This failure to translate tumor genetic profiling to the clinic is due to multiple reasons, including inefficiencies in the clinical trial process, complex tumor mutation profiles, few “druggable” targets, and a lack of predictive tools to match patients with therapies. Thus, there is a clear clinical need for accurate and timely selection tools that can maximize their clinical benefit while avoiding ineffective therapeutic interventions.

Preclinical tumor models serve as important tools for studying cancer biology, developing novel therapeutics, and informing treatment decisions (6, 7). Patient-derived organoids (PDOs) are self-organizing, 3-dimensional cultures derived from primary or metastatic tumor tissues (8). Compared with traditional models such as cell lines, genetically engineered mouse models, and patient-derived xenografts (PDXs), PDOs serve as patient-specific “avatars” that can be faster and less resource intensive drug screening tools compared with in vivo models (9–11). In addition, PDOs theoretically have a larger expansion capacity, including expansion of low frequency cell populations that may contribute to tumorigenesis, making them ideal for studying drug sensitivity (7, 12). PDOs of pancreatic ductal adenocarcinoma (PDAC) (11), colorectal adenocarcinoma (CRC) (13), and hepatocellular carcinoma (14) have been established from surgical resections and biopsies, and demonstrated that PDOs recapitulate the molecular and biological characteristics of the tumor of origin (13). In addition, emerging evidence from retrospective analysis of a small number of patients showed that PDO responses to chemotherapeutic agents are concordant with the initial responses of the PDO donor patients to the same agents (15–19). However, additional studies are needed to determine whether PDO responses to molecularly targeted therapies are predictive of patient responses (20).

In order to define the utility of PDOs as a model to predict patient responses to targeted therapies, we developed a biobank of PDOs generated from tissues obtained from surgical resections or biopsies. PDOs were developed with a high success rate from a variety of GI cancers and were characterized using genomic, transcriptomic, and immunohistochemical analyses and found to be representative of the donor tumor. PDOs were also useful for therapeutics studies, including testing for responses to chemotherapies as well as targeted agents based on their molecular profiles. Next, we demonstrated a high clinical correlation rate in drug response between our PDOs and the donor tumor. Interestingly, during our studies we found organoid culture media had a significant impact on PDO morphology, transcriptome, and therapeutic response. This indicates that media composition is a key variable in predicting donor tumor therapeutic response. Altogether, our study found that PDOs serve as a suitable model for predicting therapeutic response to chemotherapy and targeted agents of the donor tumor for multiple GI cancer types, but organoid culture media is an important variable influencing PDO response that should be considered in future translational and clinical efforts.

## Results

### GI cancer PDOs are representative of the donor tumors.

**Figure 1:**
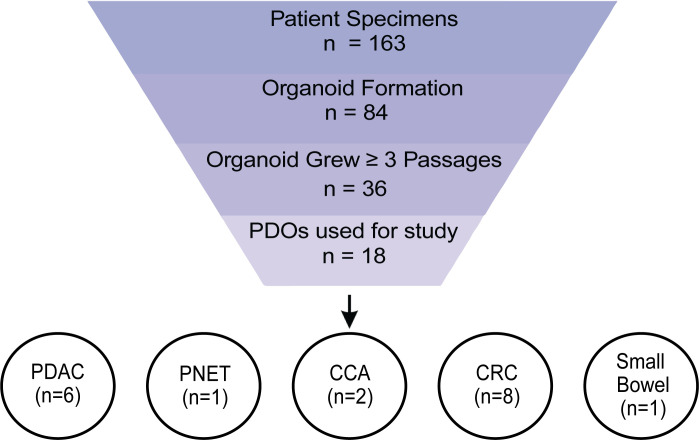
Study design. Consort diagram of patient samples and PDOs in this study. PDO, patient-derived organoid; PDAC, pancreatic ductal adenocarcinoma; PNET, pancreatic neuroendocrine tumor; CCA, cholangiocarcinoma; CRC, colorectal cancer; Small Bowel, small bowel cancer.

To evaluate the clinical value of PDO testing for cancer therapies, we developed PDOs from surgical resection, endoscopic ultrasound-guided (EUS) fine-needle biopsies (FNBs), and ultrasound-guided (US) core-needle biopsies of primary tumors and metastatic lesions of GI cancers. These tissues were collected between September 2017 and January 2021 from patients with previously or newly diagnosed GI cancers (Supplemental Table 1; supplemental material available online with this article; https://doi.org/10.1172/jci.insight.158060DS1). Over this time, we enrolled 163 patients with GI cancer, including PDAC, pancreatic acinar cell carcinoma (ACC), pancreatic neuroendocrine tumors (PNETs), cholangiocarcinoma (CCA), CRC, and small bowel cancer, with an overall success rate of 52% for organoid formation across all cancer types (Supplemental Figure 1, A and B). We generated all our GI cancer PDOs from US-biopsy except for 2 CCA samples collected by brush biopsy and the pancreatic cancer specimens, which were collected from surgical resection, EUS-FNB, or US-biopsy (Supplemental Figure 1, C and D). The primary reason for organoid formation failure following tumor cell isolation was low cellularity of the tumor specimen (Supplemental Table 2). Thirty-six PDOs grew for more than 3 passages and 18 were used for our study (Figure 1). A majority of the remaining 18 PDOs not used in this study exhibited slow proliferation or the presence of contaminating fibroblast-like cells in the PDO culture. These fibroblast-like cells appeared to outgrow or inhibit organoid growth, potentially through mesenchymal cell regulation of epithelial cells as previously described (21).

Two different types of media were utilized for pancreatic cancer organoid development over the course of this study — (serum-free) PaTOM (22) and WNT media (8, 23) — which are both commonly used for pancreatic cancer PDO generation (see Methods). All other GI cancer specimens were grown exclusively in PaTOM. We attempted to grow PDOs for 79 pancreatic cancer specimens (PDAC, ACC, and PNET) in PaTOM media, with an overall success rate of 47% (Supplemental Figure 1, E and F). To improve our success rate in PDO formation, we switched to initiating our pancreatic cancer PDOs in WNT media. Forty-two pancreatic cancer samples were initiated in WNT, with a success rate of 50% (Supplemental Figure 1, E and F). For the remainder of the study, we grew our PDAC PDOs in either PaTOM or WNT media unless otherwise noted (Supplemental Table 2). We also generated 10 PDOs from pancreatic cancer PDX models for a total of 28 PDOs utilized for either exome sequencing, RNA sequencing (RNA-seq), and/or pharmacological studies (Supplemental Table 3).

**Figure 2:**
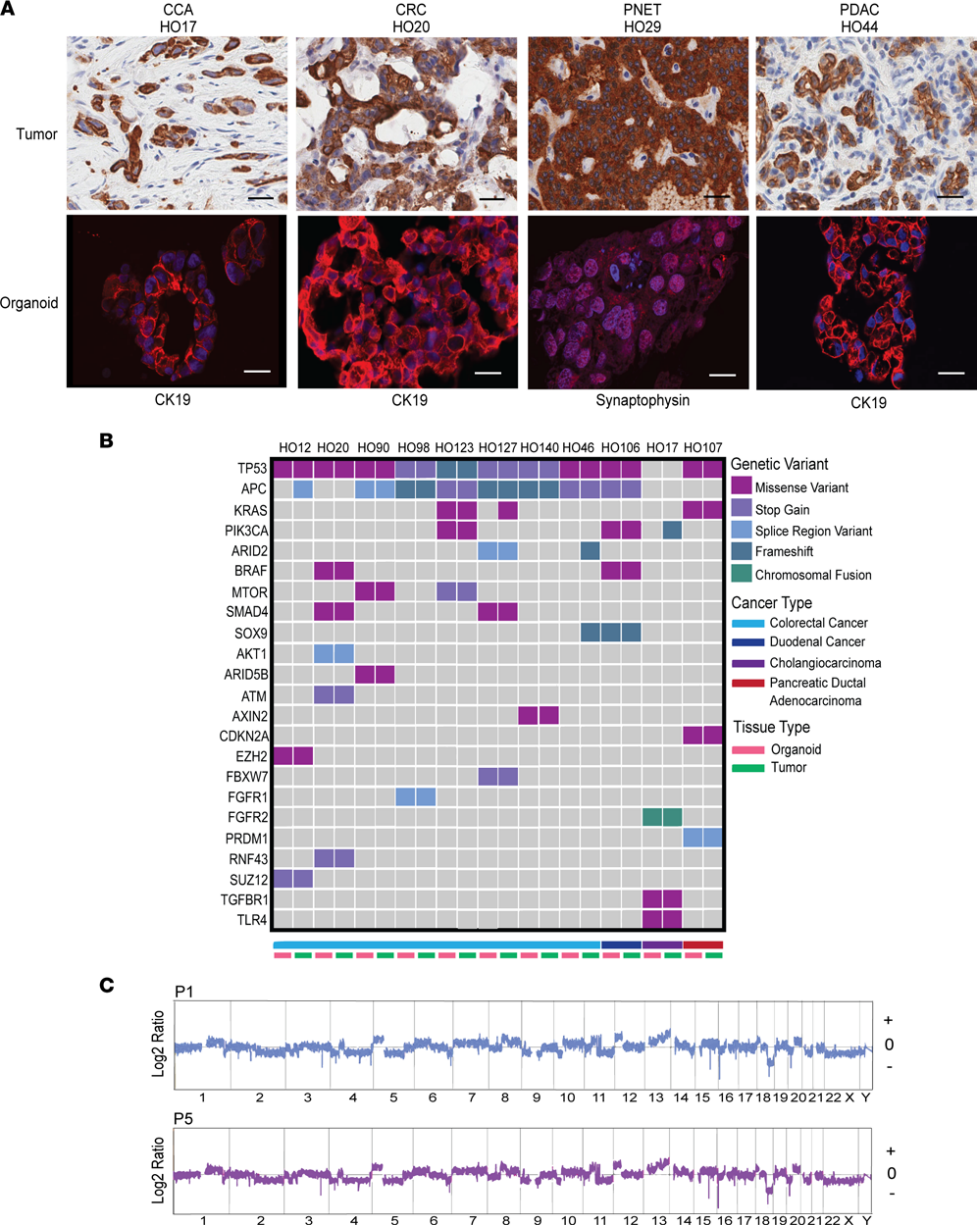
GI cancer PDOs are representative of the donor tumor. (**A**) IHC of donor tumor (scale bars: 25 μm) and confocal IF images of corresponding organoid (scale bars: 20 μm) for common surface markers for GI cancers. In organoid panels, CK19 and synaptophysin are shown in red and DAPI in blue; *n* =4. (**B**) Genetic comparison of clinically relevant variants between the PDO and the corresponding donor tumor; *n* = 11. (**C**) Copy number variant (CNV) profile of CRC PDO HO12 at passage 1 and passage 5 using CGH. The *x* and *y* axes in the CGH plots represent chromosome and position, and log_2_ ratios for each chromosome, respectively.

Similar to other studies, our PDO lines are quite heterogeneous, exhibiting a wide variety of morphological characteristics and growth rates (11, 18). H&E staining of the PDO and the corresponding primary tumor showed the PDOs retained distinct morphological features consistent with their tumor of origin and the same tumor grade between tumor and PDO (Supplemental Figure 2). We demonstrated that characteristic markers between the primary tumor tissue and corresponding PDO were similar for different cancer types (Figure 2A and Supplemental Figure 3). A novel next-generation sequencing platform, the 800 Cancer Gene Panel, developed at Mayo Clinic, was utilized to identify pathogenic mutations in 8 of the PDOs. We sequenced an additional 13 PDOs using the QIASeq Targeted DNA panel. Clinically relevant mutations identified in 11 PDOs were compared with mutations in the corresponding primary tumor (when tumor sequencing data were available) and found to be highly conserved (Figure 2B), with an 88% concordance rate between PDO and donor tumor for these mutations. Using comparative genomic hybridization (CGH), we demonstrated that the copy number variants (CNVs) of PDO were stable from passage 1 to passage 5 (Figure 2C and Supplemental Figure 4, A–C), showing that our PDOs retained the genomic landscape and mutational patterns of their primary tumor source across multiple passages.

### Genomic and transcriptomic profiling of PDOs provides insights into drug sensitivity.

Based on targetable mutations identified in the PDOs, we tested whether our models could predict response to therapy using genomic profiling. Exome sequencing of a liver biopsy from a CCA patient intrahepatic metastasis identified what we believe is a novel *FGFR2-KIF5C* chromosomal fusion, which we also detected in the corresponding PDO (HO17) (Supplemental Figure 5A). This PDO exhibited high sensitivity to the FGFR inhibitor BGJ398, and intermediate sensitivity to the multikinase inhibitor lenvatinib compared with a CCA PDO, HO42, with no FGFR aberrations (Supplemental Figure 5, B and C). We also tested BGJ398 sensitivity in 3 pancreatic cancer PDOs, including 1 (HO219) with a *FGFR1-ERLIN2* chromosomal fusion (Supplemental Figure 5D), and observed increased sensitivity to BGJ398 in HO219 compared with 2 FGFR–wild-type PDAC PDOs (Supplemental Figure 5E). This demonstrates a correlation in response to FGFR inhibition and FGFR status in our PDOs. Additional sequencing of 3 PDAC PDOs identified a PDO with a *BRCA2* pathogenic mutation (HO162), which is associated with homologous recombination deficiency (HRD) and sensitivity to PARP inhibition (Supplemental Figure 5F). We measured response to the PARP inhibitor rucaparib, and found that HO162 was more sensitive to rucaparib than 1 non-HRD PDAC PDO, showing a correlation with HRD mutation status and response to PARP inhibition in our PDOs (Supplemental Figure 5G). However, PDO HO107 was also relatively sensitive to PARP inhibition and had no known HRD-associated mutations. These results demonstrate that PDO genomic profiling was able to accurately predict therapeutic response to the FGFR inhibitor BGJ398 (including differential sensitivity to 2 inhibitors for CCA) but was less predictive for PARP inhibition.

Next, we evaluated whether the PDAC PDO transcriptome could predict response to epigenetic cancer therapies. First, we measured drug sensitivity of PDOs to the bromodomain and extraterminal domain (BET) protein inhibitor, JQ1, and the histone deacetylase inhibitor, suberoylanilide hydroxamic acid (SAHA), in 4 PDAC PDOs (Supplemental Figure 6, A and B). We compared the transcriptomes between the sensitive and resistant PDOs based on response. For both JQ1 and SAHA, the same PDAC PDO was more resistant (HO107) versus more sensitive (HO44). HO107 is mutant KRAS G12D, while HO44 is wild type, which may contribute to its resistance to JQ1 and SAHA (24, 25). Gene set enrichment analysis (GSEA) showed enrichment of genes upregulated and downregulated by KRAS activation in HO107 (Supplemental Figure 6C and Supplemental Table 4). Examination of the differentially expressed genes between the 2 PDOs identified a significant increase in mRNA expression of the transcription factor GLI2 in the resistant PDO (HO107) (Supplemental Figure 6D). It has previously been reported that pancreatic cancer cells with upregulation of GLI2 and increased expression of markers for epithelial-mesenchymal transition (EMT), which was also enriched in GSEA for HO107, are associated with JQ1 and chemotherapy resistance (Supplemental Figure 6E) (26–28). These results indicate that evaluation of transcriptomic profiles in PDAC PDOs may aid in predicting sensitives to epigenetic agents.

### Culture media affect morphology, transcriptome, and drug sensitivity of PDAC PDOs.

Next, we sought to evaluate therapeutic response of our library of pancreatic cancer PDOs to common chemotherapies. A study by Huang et al. noted that PDAC PDOs in WNT and PaTOM media showed differences in growth and differentiation status (20). Since we utilized both media in our PDAC PDOs, we evaluated their impact on PDO response to chemotherapy. To measure this effect, we grew 5 PDOs in both PaTOM and WNT media and compared morphology, therapeutic response, and transcriptomes. Of note, we initiated all these PDOs in PaTOM media before switching to WNT media, which could have affected initial cell selection during initiation.

**Figure 3:**
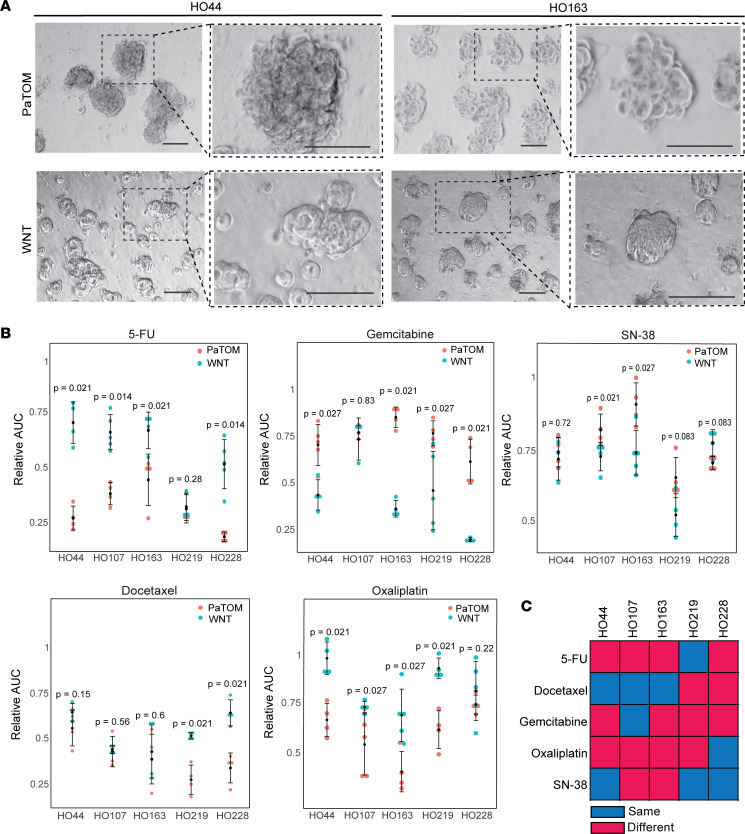
PDOs grown in 2 different culture media exhibit differential response to chemotherapy. (**A**) Brightfield images of PDAC PDOs HO44 and HO163 grown in WNT and PaTOM media at 2 magnifications (scale bars: 100 μm). (**B**) Relative AUC values for 5 PDAC PDOs in response to 5-FU, gemcitabine, SN-38, docetaxel, and oxaliplatin grown in PaTOM and WNT media. Each dot represents the relative AUC for 1 replicate for each PDAC PDO derived from the line graphs in Supplemental Figure 8. *P* values indicate level of significance using Wilcoxon’s rank sum test between AUCs of PDOs grown in PaTOM versus WNT media; *n* = 5. (**C**) Summary of 5 PDAC PDO responses in PaTOM and WNT to 5 chemotherapies. Red indicates response was significantly different (*P* ≤ 0.05) between PaTOM and WNT for the same PDO. Blue indicates the response was not significantly different (*P* > 0.05) between media.

We observed distinct morphological and growth rate differences in PDOs between media, similar to previously reported findings (Figure 3A and Supplemental Figure 7) (20). We then compared drug response of our PDOs to common PDAC cancer therapies, including fluorouracil (5-FU), gemcitabine, SN-38 (irinotecan active metabolite), docetaxel, and oxaliplatin in both media (Figure 3B and Supplemental Figure 8). We demonstrated significant differences in response between PDOs grown in WNT and PaTOM media in each of these drugs (Figure 3C). Interestingly, a majority of PDOs grown in PaTOM media were significantly more sensitive to 5-FU and oxaliplatin, while PDOs grown in WNT were significantly more sensitive to gemcitabine (Figure 3B and Supplemental Figure 9, A and B). Two of 5 PDOs grown in WNT were also significantly more sensitive to SN-38, while 2 of 5 PaTOM PDOs were significantly more sensitive to docetaxel (Figure 3B). This indicates that PDO culture media influence therapeutic response for multiple chemotherapies.

**Figure 4:**
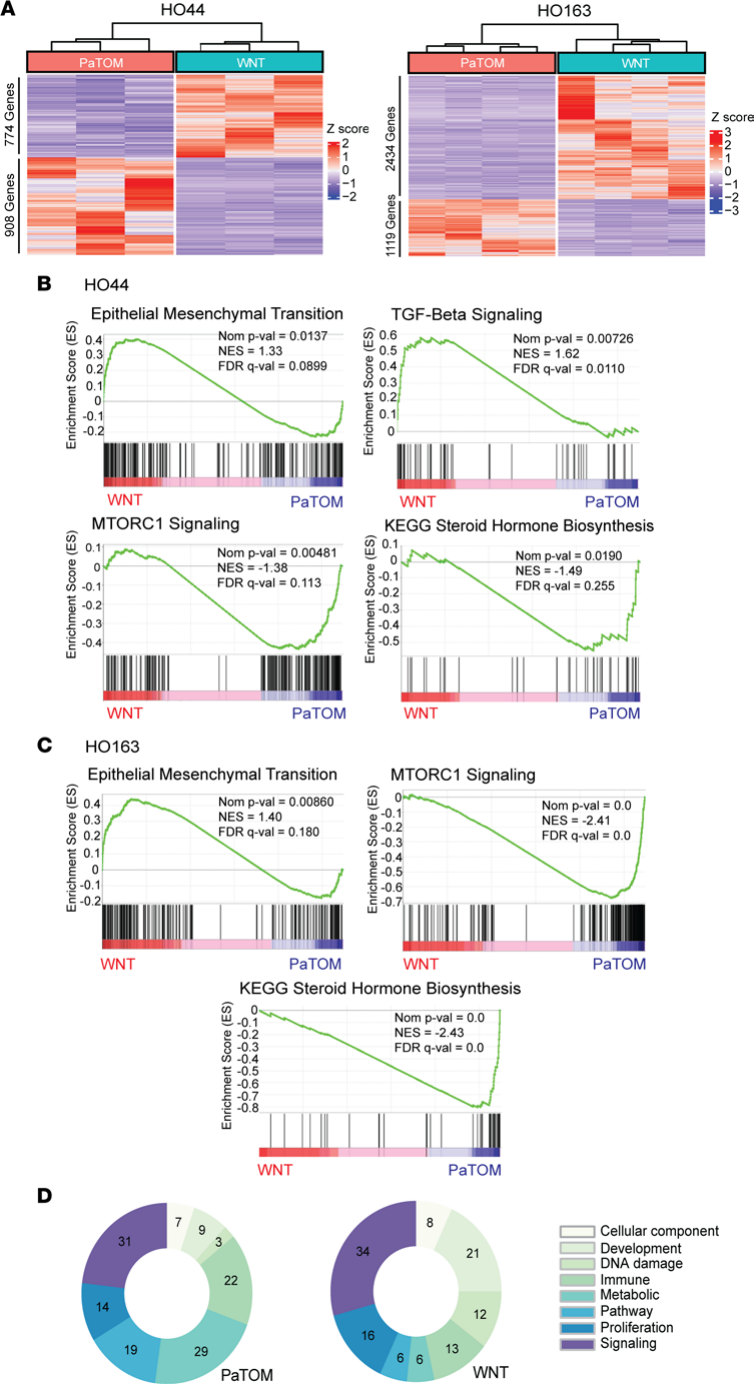
PDOs grown in 2 different culture media show distinct transcriptomic signatures. (**A**) Heatmaps showing clustering by differentially expressed genes for HO44 and HO163 PDOs cultured in WNT and PaTOM media with base mean ≥ 1, |fold change| > 2, and *P* value < 0.05; *n* = 2. (**B**) Gene sets enriched in PDAC PDO HO44 in WNT and PaTOM media using GSEA for Hallmark and KEGG gene sets. (**C**) Gene sets enriched in PDAC PDO HO163 in WNT and PaTOM media using GSEA for Hallmark and KEGG gene sets. NES, normalized enrichment score. (**D**) Summary of RNA-seq expression data for all 5 PDAC PDOs in WNT and PaTOM using GSEA Hallmark Gene Set categories; *n* = 5.

To elucidate this difference in response, we evaluated transcriptomic data from these 5 PDOs in both media. Differential gene expression analysis of RNA-seq data demonstrated a high level of variance between WNT and PaTOM media for each PDO (Supplemental Figure 10A) and distinct clustering of significantly differentially expressed genes between media (Figure 4A and Supplemental Figure 10B). GSEA of PDO HO44 showed enrichment in WNT media for EMT markers and TGF-β signaling (Figure 4B and Supplemental Table 5G). Increased TGF-β signaling is associated with EMT and tumor resistance to 5-FU and oxaliplatin (29–31). PaTOM media showed an increase in expression of MTORC1 signaling and steroid hormone biosynthesis (Figure 4B and Supplemental Table 5, G and H). PaTOM medium contains hydrocortisone, which is part of the cholesterol pathway for steroid biosynthesis. Also, PaTOM contains insulin, which has been demonstrated to induce the MTOR signaling pathway (32, 33). Upregulated MTORC1 signaling is associated with increased resistance to gemcitabine in pancreatic cancer cells (34, 35). GSEA for PDO HO163 revealed enrichment of similar gene sets (Figure 4C and Supplemental Table 5, A and B). In fact, MTORC1 signaling was upregulated in all 5 PDOs grown in PaTOM media and steroid biosynthesis was enriched in 4 of 5 PDOs. WNT medium was enriched for EMT in all 5 PDOs, TGF-β signaling in 3 PDOs, and E2F targets in 3 PDOs (Supplemental Table 5). E2F transcription factors are associated with chemotherapy resistance (36), potentially through promotion of cancer stem cells (37, 38). Global analysis of RNA-seq data using GSEA Hallmark Gene Set process categories showed PDOs grown in WNT were enriched with genes associated with development and DNA damage, while PDOs in PaTOM media were enriched in genes associated with metabolism, immunity, and pathways (Figure 4D, Supplemental Figure 11, A–E, and Supplemental Table 5K) (39).

Of note, this difference in response between media was not only observed in chemotherapies. We evaluated the sensitivity of 12 pancreatic cancer PDOs to common targeted therapies in PaTOM or WNT media (these PDOs were not grown in both media), including BGJ398, JQ1, pemigatinib, rucaparib, and SAHA (Supplemental Figure 12, A–E). PDAC PDOs grown in PaTOM exhibited significantly higher sensitivity to rucaparib and SAHA than those grown in WNT media, and we saw less variability in response for PDOs grown in WNT media to targeted therapies (Supplemental Figure 12F). For example, we identified 4 PDAC PDOs grown in WNT with HRD-associated pathogenic variants (Supplemental Figure 12G) and saw no significant differences in response to PARP inhibition relative to mutational status (Supplemental Figure 12H). This indicates media may also influence therapeutic response to targeted therapy. However, since the PDOs grown in WNT and PaTOM media were from different patients (not the same PDO grown in both media), our results might be affected by unique properties of the individual patients.

Since pancreatic cancer subtype can predict therapeutic response, we evaluated whether PDO culture media influenced transcriptomic subtype. There is currently a consensus that PDAC can be divided into classical and basal transcriptional subtypes, with the basal subtype having a worse prognosis (40–42). Using a previously described bioinformatics pipeline (40), we analyzed RNA-seq data from these 5 PDAC PDOs over multiple passages in WNT and PaTOM media and clustered them as either classical or basal subtype (Supplemental Figure 13). We found that 4 of 5 PDOs classified as the same subtype in WNT and PaTOM media. One PDO, HO228, was found to have basal subtype in WNT media and classical subtype in PaTOM. There was no difference in subtype between passages for all the PDOs (Supplemental Figure 13).

**Figure 5:**
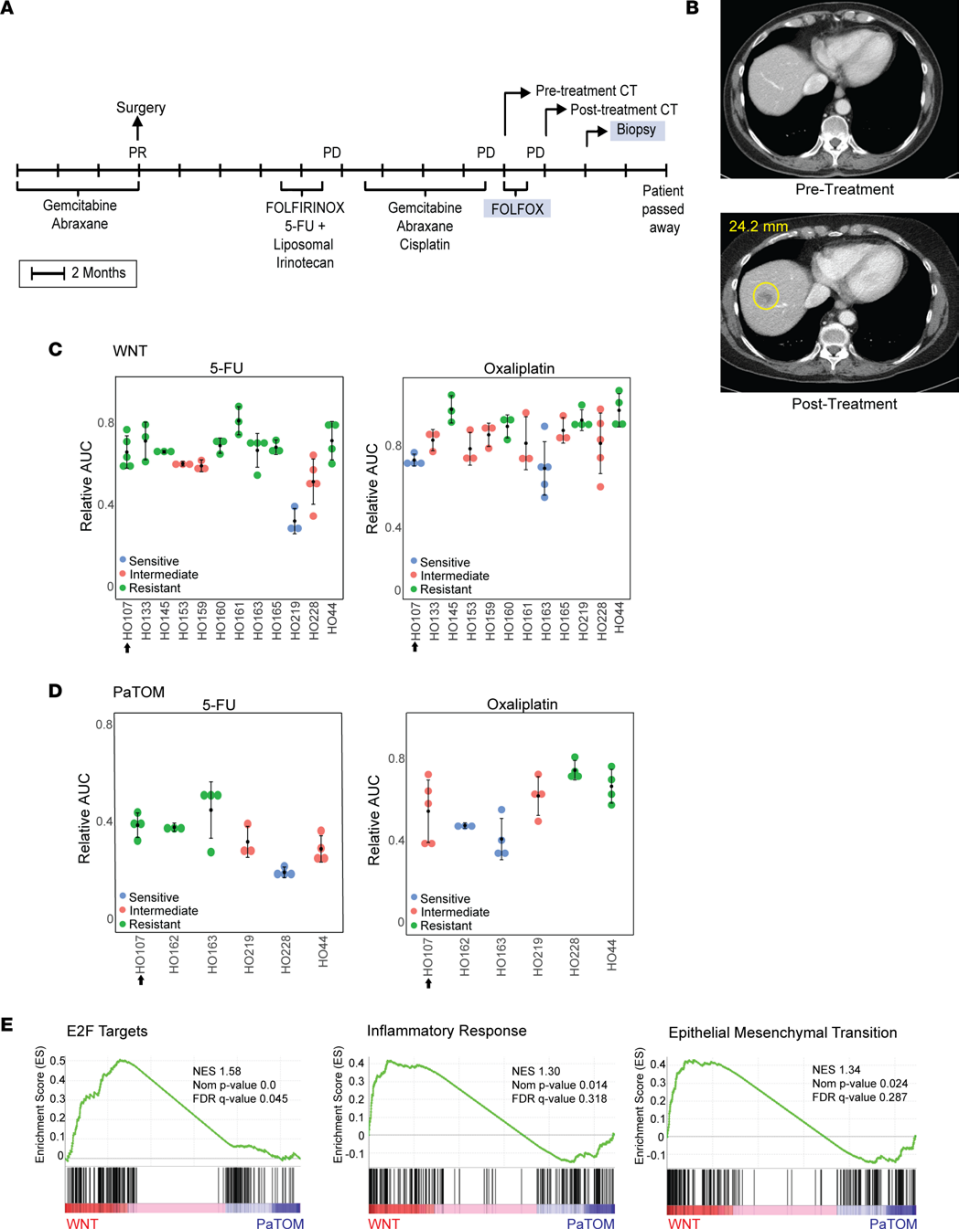
The effect of PDO culture media on clinical correlation: a case study. (**A**) Donor patient treatment history for PDO HO107. After surgery, this patient received a single dose of FOLFIRINOX (folinic acid + 5-FU + irinotecan + oxaliplatin) followed by 5-FU and liposomal irinotecan. (**B**) CT scans of donor patient for PDO HO107 before and after treatment with progressive disease (PD), where a metastatic liver lesion of 24.2 mm developed following treatment with FOLFOX. (**C**) Relative AUC values for PDO HO107 in response to 5-FU and oxaliplatin in WNT media. Each dot represents the relative AUC for 1 replicate derived from the line graphs in Supplemental Figure 14A; *n* = 12. (**D**) Relative AUC values for PDO HO107 in response to 5-FU and oxaliplatin in PaTOM media. Each dot represents the relative AUC for 1 replicate derived from the line graphs in Supplemental Figure 14B; *n* = 6. The data in this figure for HO17, HO44, HO163, HO219, and HO228 were derived from Supplemental Figure 8. PDO response was determined as intermediate, sensitive, or resistant using Jenks Natural Breaks. The arrow next to the PDO number indicates the PDO response corresponding to the patient tumor response in the CT scan. (**E**) GSEA using Hallmark gene sets showing enriched gene sets in PDAC PDO HO107 in WNT media. NES, normalized enrichment score.

Since we utilized 2 media for our PDAC PDOs, we were interested in determining which media best matched the donor tumor clinical response. For this comparison, we evaluated drug sensitivity of a PDAC PDO from a metastatic liver biopsy, HO107, in both WNT and PaTOM media. The donor patient received FOLFOX (folinic acid + 5-FU + oxaliplatin) treatment prior to biopsy and showed progressive disease (PD) (Figure 5, A and B). HO107 was treated with oxaliplatin and 5-FU in WNT and PaTOM media and response was categorized using the Jenks Natural Breaks algorithm as sensitive, intermediate, or resistant based on the average AUC of HO107 and additional PDAC PDOs (see Methods) (Supplemental Figure 14, A and B). In WNT media, HO107 was categorized as resistant to 5-FU and sensitive to oxaliplatin, which was a mismatch with patient response (Figure 5C). In PaTOM media, HO107 was resistant to 5-FU and intermediate to oxaliplatin (considered nonsensitive), which matched the patient response (Figure 5D). These data indicate that this PDO better matched patient tumor response when grown in PaTOM media. Like other organoids grown in both media, this PDO was more resistant to oxaliplatin and 5-FU in WNT media compared with PaTOM (Supplemental Figure 9B). HO107 also had a similar gene signature associated with oxaliplatin and 5-FU resistance seen in the other WNT PDOs, including increased expression of EMT, E2F targets, and inflammatory response according to Hallmark GSEA (Figure 5E and Supplemental Table 5I).

We assessed an additional PDAC PDO, HO145, for clinical correlation in WNT media. The donor patient was treated with docetaxel following their metastatic liver biopsy and showed PD (Supplemental Figure 14C). The PDO was sensitive to docetaxel in WNT media, which was a mismatch with patient response (Supplemental Figure 14D). We were unable to test drug response for this PDO in PaTOM media since PDOs initiated in WNT media did not grow when switched to PaTOM. Based on these mismatches in response in WNT media, we used only PaTOM media for our clinical correlation studies.

### PDO therapeutic agent sensitivities correlate with patient tumor response.

**Figure 6:**
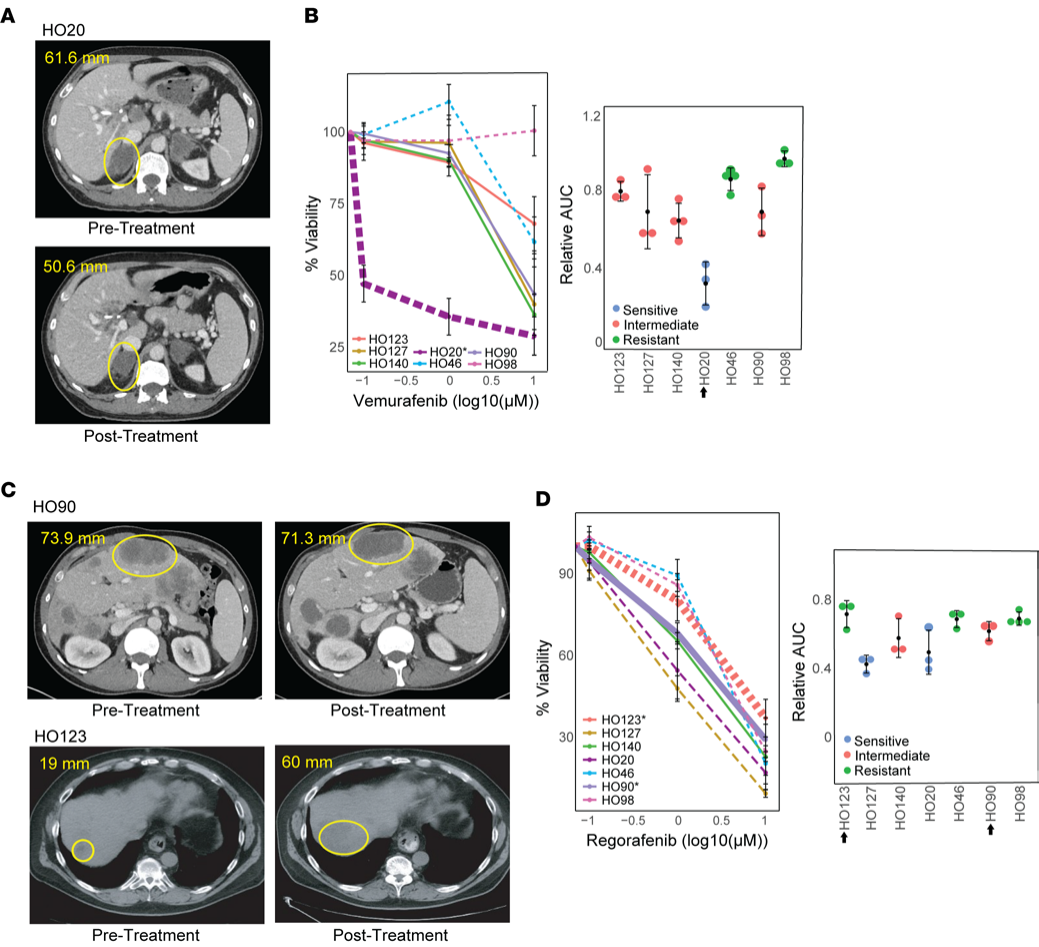
PDO therapeutic agent sensitivity correlates with patient tumor response. (**A**) CT scans of donor patient for PDO HO20 before and after treatment with partial response (PR), where the lesion decreased from 61.6 mm to 50.8 mm after 2 cycles of treatment with vemurafenib. (**B**) Percentage cell viability and relative AUC values in response to vemurafenib for 7 CRC PDOs, showing that PDO HO20 is sensitive to vemurafenib; *n* = 7. (**C**) CT scans of donor patients before and after treatment showing stable disease (SD) in 1 lesion (HO90) and progressive disease (PD) in 1 lesion (HO123) following treatment with regorafenib. (**D**) Percentage cell viability and relative AUC values in response to regorafenib for 7 CRC PDOs, showing that PDO HO90 is intermediate and HO123 is resistant to regorafenib; *n* = 7. For line graphs, dashed lines indicate sensitive, solid lines indicate intermediate, dotted lines indicate resistant, categorized using the Jenks Natural Breaks algorithm. The HO number with an asterisk and the thickest line in the line graphs indicate PDO response corresponding to the patient tumor response shown in the CT scan. The arrow next to the HO number in the relative AUC graph indicates the PDO corresponding to the donor tumor.

We next defined the value of PDOs in predicting the donor patients’ clinical response to anticancer agents in cases where we had both patient and PDO treatment responses. Patient response was categorized as PD, partial response (PR), or stable disease (SD) using Response Evaluation Criteria in Solid Tumors (RECIST) (Supplemental Table 6). PDOs were treated with the same therapy(s) as the donor tumor and PDO response was categorized using the Jenks Natural Breaks algorithm or published IC_50_ values (see Methods). Of the 11 cases, 9 (82%) demonstrated PDO responses that were consistent with the donor patient’s response (Table 1). One case, a CRC patient with mutant BRAF V600E, had PR to 2 cycles of treatment with the BRAF inhibitor, vemurafenib, and the anti-EGFR monoclonal antibody, cetuximab (HO20) (Figure 6A). The corresponding PDO showed no significant response to single-agent treatment with cetuximab (Supplemental Figure 15N), but its growth was inhibited by vemurafenib (Figure 6B), consistent with the PR of the patient. In a second case, a donor patient was treated with regorafenib and had SD in 1 lesion (HO90) (Figure 6C). An additional donor patient was treated with regorafenib and had PD in 1 lesion (HO123) (Figure 6C). The corresponding PDOs showed intermediate regorafenib sensitivity for HO90 and regorafenib resistance for HO123, consistent with the patients’ response (Figure 6D).

Two of 11 PDOs did not match donor patient clinical response (Table 1). In the first case, the donor CCA patient was treated with the nucleoside analog TAS-102 (thymidine phosphorylase inhibitor), resulting in PD (HO42) (Supplemental Figure 15D). The PDO response to TAS-102 had an IC_50_ of 303 nM (Supplemental Figure 15E). According to Vlachogiannis et al., an IC_50_ of less than 500 nM indicates sensitivity to TAS-102 (19). In the second case, a donor CRC patient was treated with capecitabine (an oral prodrug of 5-FU), oxaliplatin, and panitumumab, resulting in PR (HO140) (Supplemental Figure 15M). The PDO from this patient was resistant to 5-FU and oxaliplatin and showed intermediate sensitivity to cetuximab (Supplemental Figure 15, K, L, and N). We tested a combination of 5-FU, oxaliplatin, and cetuximab in this PDO and did not see a significant increase in sensitivity (data not shown). Further information on the remaining cases is described in Supplemental Table 6. CT scans and PDO responses can be found in Supplemental Figure 15. Together, these studies demonstrate that for a majority of cases, PDO sensitivity to therapeutic agents matched the patients’ response to the same agent(s).

## Discussion

Our prospective study demonstrates the feasibility of growing PDOs from several different types of GI cancer and specimen sources using a single protocol. The success rate of establishing PDOs from GI malignancies varied widely, as seen in previous studies (11, 43). Notably, CRC had a higher rate of PDO formation compared with PDAC or CCA. This is presumably due to differences in the cellular and genetic makeup of individual primary tumors and intrinsic properties (e.g., numbers of cancer stem cells) of different types of tumor tissue. Other factors such as tumor specimen size, cellularity, and desmoplastic status can significantly affect PDO establishment (18, 19, 43). A standardized process for measuring tumor cellularity of the specimen during collection would increase the success rate for PDO development. Also, individualizing organoid culture media to target driver mutations in the patient tumor genetic profile, such as EGF-depleted media to enrich for KRAS mutants, may increase PDO formation and eliminate nontumor “contaminants” that may inhibit PDO growth (44).

Few studies have validated the ability of PDOs to predict response to targeted cancer therapies in the clinical setting. While genomic profiling of the donor tumor should match patient sensitivity to targeted therapy, molecular mutations have not been the only determinant for response. For example, we showed that PDOs can predict response to FGFR inhibition based on genomic profiling, as previously reported (45–47), but we saw no response to rucaparib in PDOs with pathogenic HRD mutations. Previous studies have reported that HRD mutation status is not the sole predictor of response to rucaparib in pancreatic cancer (48, 49). In this regard, we demonstrated that PDOs may serve as a tool for predicting response in cases when the genomic profile does not match tumor response. In addition to genomic profiling, review of the PDO transcriptome can help identify therapeutic sensitivity in the donor tumor, as shown in this study. Nicolle et al. observed that pancreatic cancer transcriptomic signatures can predict gemcitabine sensitivity and improved overall disease-free survival in patients due to a broader spectrum of disease phenotypes (50). Analysis of the transcriptome to identify cancer therapy sensitivities would be particularly beneficial in patients with advanced PDAC since it can be performed quickly. However, since our study was performed in only a small number of PDAC PDOs, transcriptomic evaluation for predicting therapeutic response should be further investigated using a larger subset of PDOs.

As we demonstrated, PDO culture medium was shown to have a significant impact on PDO transcriptome, morphology, and therapeutic response. Similar to a previous study by Huang et al., we observed an enrichment of markers associated with dedifferentiation in WNT media PDOs, which may alter the PDO phenotype (20). In addition, we found that PDOs grown in WNT media were typically more resistant to chemotherapy and targeted therapies and were enriched for E2F targets, EMT, and TGF-β pathways relative to PDOs grown in PaTOM, which have been associated with increased drug resistance. However, this increased resistance was not universal. Interestingly, PDOs in PaTOM were more resistant to gemcitabine and SN-38. Further studies are needed to understand the effect of media on transcriptomic subtype and drug response, such as comparing transcriptomes between the donor tumor and PDO in both media.

While we demonstrated better correlation between donor tumor and 1 PDO in PaTOM media, it will take additional correlation studies to determine the best media composition when predicting tumor response. In addition, although we observed a mismatch in response to docetaxel between the donor tumor and its PDO (HO145) in WNT media, we must note that the donor patient was treated with oradoxel (oral docetaxel). Since the drug was administered to the patient orally this may have affected drug absorption and/or metabolization and response. Importantly, several studies have already demonstrated that PDOs grown in WNT media correlate with patient tumor response in GI cancer (18, 19) and PaTOM (51). However, our study shows that culture media ingredients must be considered when performing correlative drug studies in PDOs and the appropriate media may vary based on the therapy(s) being tested. In our correlative study using PaTOM media, PDO and donor tumor clinical drug response matched in 9 of 11 cases in PaTOM, with a mismatch in 2 cases. In 1 case, the CCA donor patient was treated in a clinical trial with TAS-102 and showed PD, while the corresponding PDO (HO42) was sensitive. This patient experienced anemia and biliary obstruction during treatment, which may have affected tumor response. In addition, the tumor microenvironment is complex and PDOs represent a relatively pure tumor cell population, which could modify cancer cell responses. This issue could be addressed through coculture of PDOs with nontumor cells (52). Last, 9 out of 11 of the PDO/patient cases evaluated were from patients who had received 1 to 4 previous rounds of chemo- or targeted therapy, which could lead to increased drug resistance to other cancer therapies.

With further standardization, PDOs could be a powerful tool for personalizing cancer therapy. However, PDO development, including culture media conditions, and testing procedures need to be standardized to truly impact the care of patients with advanced disease. First, clinical trials including large patient cohorts receiving identical therapeutic agents need to be accompanied with standardized tissue collection and PDO development and drug testing methodologies. Finally, development of assays requiring a smaller number of cells for drug testing need to be clinically validated to aid in quick clinical treatment decisions. With these steps, PDOs have enormous potential to positively impact care for patients with GI cancers.

## Methods

### Tumor specimen processing and PDO culture conditions.

Tissue samples from patient or PDX tumors were used for the preparation of PDOs, including surgical tumor resections, EUS-FNB of the primary tumor, or US-biopsy of tumor metastases. The fresh tumor specimens were immediately placed in resuspension media containing DMEM (Corning, 10-013-CV), 1% Penicillin-Streptomycin-Amphotericin B Solution (ATCC, PCS-999-02), and 1% bovine serum albumin (BSA) (Equiteck-Bio, BAL62-0500), stored on ice, and brought to the processing lab. Specimens were stored for a maximum of 24 hours at 4°C prior to processing. Tissue was minced using a no. 22 surgical blade (Bard-Parker, 371122) in 0.5 mL of resuspension media, enzymatically digested using a human Tumor Dissociation Kit (Miltenyi Biotec, 130-095-929) in a gentleMACS Octo Dissociator with Heaters (Miltenyi Biotec), filtered through a MACS SmartStrainer (100 μm) filter (Miltenyi Biotec, 130-098-463), and cells were plated in 1 well of 12-well flat-bottom cell culture plate (Corning, 3513) coated with 100 μL of Corning Matrigel Growth Factor Reduced (GFR) Basement (Corning, 354230) in 1 of 2 types of organoid culture media: WNT media (8, 23) or PaTOM (serum-free media) (22). WNT medium contains 50% L-WRN–conditioned media (Wnt3a, R-spondin, and Noggin) (53), 50% Advanced DMEM/F12 (Gibco, 12634-010), 1× HEPES (Gibco, 15630-080), 1× GlutaMax (Gibco, A12860-01), 1× N2 Supplement (Gibco, 17502048), 1× B27 Supplement (Gibco, 12587-010), 50 ng/mL EGF (R&D Systems, 236EG200), 3 μM SB202190 (Selleckchem, S1077), 500 nM A-83-01 (Selleckchem, S7692), 1 mM *N*-acetylcysteine (Sigma-Aldrich, A9165), 10 mM nicotinamide (Sigma-Aldrich, N3376), 10 nM gastrin I (Sigma-Aldrich, G9020), 100 ng/mL FGF10 (Peprotech, 100-26), 100 μg/mL Primocin (Invivogen, ANT-PM-1), and 1% Penicillin-Streptomycin-Amphotericin B Solution. PaTOM medium contains DMEM plus GlutaMax (Gibco, 10564-011), 0.1% Penicillin-Streptomycin-Amphotericin B Solution, 0.25 μg/mL hydrocortisone (Sigma-Aldrich, H0888), 1% B27 (Gibco, 12587-010), 50 μg/mL L-ascorbic acid (Sigma-Aldrich, A92902), 20 μg/mL insulin (Sigma-Aldrich, I9278), 100 ng/mL FGF2 (R&D Systems, 233-FB), and 100 nM all-*trans* retinoic acid (Sigma-Aldrich, R2625). PDO culture medium consisted of WNT or PaTOM media plus 10 μM Y-267632 (ROCK inhibitor) (Selleckchem, S1049) and 5% Matrigel. If red blood cells (RBCs) were present following cell isolation, RBCs were lysed using RBC Lysis Solution (10×) (Miltenyi Biotec, 130-094-183) before plating. Once the PDO was established, organoids were passaged every 2–3 weeks by adding 0.5 mL of digestion media containing DMEM (Corning, 10-013-CV), 1% Penicillin-Streptomycin-Amphotericin B Solution, 1 mg/mL Collagenase/Dispase (Sigma-Aldrich, 10269638001) and incubating for 2–3 hours at 37°C followed by dissociation in 0.5 mL TrypLE (Gibco, 12604013) for 1–2 minutes in a 37°C waterbath to obtain single cells and replated in Matrigel-coated dishes. Aliquots of cells were frozen in Cryostor cell preservation media (Sigma-Aldrich, C2874) with 10 μM Y-267632 to supplement our PDO biobank. RNA and DNA were collected during passaging for transcriptomic and genetic analysis. Mouse cells were depleted from PDX-derived PDOs following initiation using the Mouse Cell Depletion Kit (Miltenyi Biotec, 130-104-694).

### Cell lines.

L-WRN cells used to generate Wnt3a-, R-spondin–, and Noggin-conditioned media were a gift from Brooke Druliner and Lisa Boardman (Division of Gastroenterology, Mayo Clinic).

### Drug study protocol.

For drug studies, cells isolated from PDOs by trypsinization were seeded at approximately 1,000–3,000 cells per well in 96-well flat-bottom plates (Corning, 3595) precoated with 10 μL of Corning Matrigel GFR Basement (Corning, 354230). Before seeding, cells were filtered through a MACS SmartStrainer (100 μm) filter (Miltenyi Biotec, 130-098-463) to remove large organoids. Plates were incubated overnight, and drug was added the next day (5–10 wells per condition). Each well contained an equal molar concentration of diluent. Plates were incubated for 5–7 days. Drug and media were refreshed every 2–3 days. Plates were imaged using the Celigo Image Cytometer (Nexcelom) on day 0 and days 5–7. Cell viability was measured using the MTT proliferation assay. MTT solution (5 mg/mL stock) was added to each well (1:10 dilution) and incubated for 2–3 hours at 37°C. Media were removed and cells were lysed in solvent (10% SDS in 0.01N HCl) and incubated for 1–2 hours at 37°C. Absorbance was measure by a spectrophotometer (Molecular Devices SpectraMax M3) at 540 nm to determine cell viability and normalized relative to the control wells. The means of a minimum of 3 biological replicates were expressed relative to the control as percentage cell viability. AUC was calculated based on relative cell viability using the trapezoid rule and was normalized to the maximum AUC value in Excel. Chemotherapy drugs used for this study included 5-FU (Selleckchem, S1209), docetaxel (Selleckchem, S1148), gemcitabine (Selleckchem, S1714), oxaliplatin (Selleckchem, S1224), SN-38 (irinotecan metabolite) (Selleckchem, S4908), TAS-102 (2:1 trifluridine [Selleckchem, S1778]/tipiracil [Selleckchem, S3731]), and paclitaxel (abraxane) (Selleckchem, S1150). Targeted cancer therapies used for this study included BGJ398 (infigratinib) (Selleckchem, S2183), JQ1 (Selleckchem, S7110), pemigatinib (Incyte Corporation, INCB054828-7), rucaparib (Selleckchem, S1098), SAHA (vorinostat) (Selleckchem, S1047), lenvatinib (Selleckchem, S1164), erlotonib (Selleckchem, S1023), vemurafenib (Selleckchem, S1267), cetuximab (Selleckchem, A2000), eFT-508 (tomivosertib) (Selleckchem, S8275), bozitinib (ProbeChem, PC-35732), and regorafenib (Selleckchem, S1178). Drugs were prepared according to the manufacturers’ instructions.

### Genetic profiling of primary tumors and PDOs.

DNA was extracted using the DNeasy Blood and Tissue Kit (Qiagen, 69504). Eight of the PDOs were sequenced using the Onco-seq Panel (HO12, HO17, HO20, HO29, HO44, HO90, HO98, and HO106), a novel 800-gene-focused exome panel developed at the Mayo Clinic. Samples were prepared for sequencing using the Agilent SureSelect XT Low Input Target Enrichment for Illumina Paired-End Multiplexed Sequencing Protocol. Two hundred nanograms of input DNA was used for each library preparation for both DNA target capture and Agilent OneSeq Hi Res CNV Backbone. Once library preparation was completed, the DNA target reactions were combined at equimolar ratios in one pool, and backbone libraries were combined at equimolar ratios in a second pool. These pools were then combined at a 2:1 ratio (100 μL of DNA target pool with 50 μL of backbone pool) to form the final loading pool. This pool was loaded onto a NovaSeq S2 flow cell following the NovaSeq 6000 System Guide.

Thirteen of the tumor samples were sequenced using the QIAseq Targeted DNA Panel (HO42, HO46, HO107, HO123, HO127, HO133, HO140, HO145, HO153, HO159, HO160, HO161, and HO165). Libraries were prepared following the manufacturer’s protocol (Qiagen). Briefly, 100 ng of genomic DNA was enzymatically fragmented, end-repaired, and A-tailed in a single, multiple-reaction step. After the multi-enzymatic reaction, an Illumina specific adapter that includes a unique molecular barcode and sample index was ligated to the 5′ end of the fragmented, end-repaired DNA. Samples were then subjected to a size selection and a purification step using QIAseq beads (Qiagen). Enriched libraries were purified using QIAseq beads and further amplified through a universal PCR reaction in which Illumina specific adapters were added and library yield was increased. The universal amplification reaction was purified, and the final library quantified by Qubit (Invitrogen), Fragment Analyzer Standard Sensitivity NGS Fragment Analysis kit (Advanced Analytical), and qPCR-KAPA Library Quantification Kit (Roche). Targeted DNA libraries were sequenced at 12 samples per lane using the Illumina cBot and HiSeq 3000/4000 PE Cluster Kit. The flow cells were sequenced as 150 × 2 paired-end reads on an Illumina HiSeq 4000, with the QIAseq Read 1 Primer custom sequencing primer, using the HiSeq 3000/4000 sequencing kit and HCS v3.3.52 collection software. Base calling was performed using Illumina’s RTA version 2.7.3. Reads from PDO sequence data for both panels were aligned against the same Hg19 reference using bwa-mem (54). Variants were called using Mutect2 software. Variants were filtered and annotated using Ingenuity Variant analysis software (Qiagen) with default filters. Pathogenic and likely pathogenic variants that were detected with a variant allele frequency of at least 5% were considered for further analysis. Detected candidate variants were plotted using OncoPrint (55). PDO variants were compared to variants found in the donor tumor sequence by the Tempus Targeted Panel, when available.

### CGH (CNVs).

DNAs were digested for 30 minutes with DNase I prior to Klenow-based labeling. In each case, 1 μL of 10× DNase I reaction buffer and 2 μL of DNase I dilution buffer were added to 7 μL of DNA sample and incubated at room temperature and then transferred to 70°C for 30 minutes to deactivate DNase I. Sample and reference templates were then labeled with Cy-3 dUTP and Cy-5 dUTP, respectively, using a BioPrime labeling kit (Invitrogen) according to our published protocol (56). All labeling reactions were assessed using a Nanodrop assay (Nanodrop) prior to mixing and hybridization to CGH arrays (Agilent Technologies) for 40 hours in a rotating 65°C oven. All microarray slides were scanned using an Agilent 2565C DNA scanner and the images were analyzed with Agilent Feature Extraction version 11.0 using default settings. The CGH data were assessed with a series of QC metrics then analyzed using an aberration detection algorithm (ADM2) (57).

### Transcriptomic analysis.

RNA was extracted using the RNeasy Micro Kit (Qiagen, 74004). At the Robarts sequencing facility (Robarts Research Institute, London, Ontario, Canada), the quality of RNA was assessed using the Agilent 2100 Bioanalyzer, followed by RNA reduction, creation of indexed libraries, and sequenced on the Illumina NextSeq. Libraries were sequenced using Illumina NextSeq High Output 75 cycle sequencing kits for single-end sequencing. RNA-seq FASTQ files were aligned using STAR version 2.7.8a (Galaxy) to the human reference genome GRCh38. Gene-level counts were obtained using HTseq-count v.0.9.1 (Galaxy). Differential gene expression analysis was performed with R package DESeq2 version 2.11.40.7 (RStudio). Genes with a base mean of 1 or greater, log_2_(fold change) greater than 1 or less than –1, and an adjusted *P* value of less than 0.05 were considered significantly differentially expressed. GSEA (http://software.broadinstitute.org/gsea/index.jsp) was performed using the Hallmark and KEGG gene sets. Heatmaps were created using the R package ggplot2 version 3.3.3. PDAC transcriptomic subtyping was performed using a subset of genes from Moffitt et al. (40) to cluster the samples with R package ConsensusClusterPlus version 1.52.0 (PMID 20427518).

### Immunofluorescence, H&E, and immunohistochemistry.

PDOs were grown in 2-well chamber slides (Nunc, 154526) for paraffin block preparation. Once the PDOs were ready for embedding, organoids were fixed in 4% paraformaldehyde (PFA) (Electron Microscopy Sciences, 15710) for 2 hours at room temperature. Following fixation, PFA was aspirated and the chamber slides were washed with deionized water. After the chamber slides were dissembled, a no. 22 surgical blade (Bard-Parker, 371122) was used to scrape the PDOs in Matrigel into cryomolds (Tissue-Tek, 4565). Four hundred microliters of Histogel (Thermo Fisher Scientific, HG-4000-012) was added to each cryomold to completely envelope the organoids. The Histogel blocks were then transferred to tissue cassettes and placed in 10% formalin (Sigma-Aldrich, HT501128) for 16–20 hours. The cassettes were transferred to a container with eosin Y solution 0.5% alcoholic (Sigma-Aldrich, 1.02439) for 1–2 days. Next, cassettes were stored in 70% ethanol until embedding in paraffin. Paraffin embedding was performed using standard procedures for clinical specimens. Sectioning was performed using a lab microtome (Leica RM2125) at 5 μm. The sections were dried in an incubator at 40°C overnight. The slides were immersed in fresh xylene bath twice, and then in 1:1 xylene/ethanol.

The slides were rehydrated with a series of 100%, 95%, 75%, and 50% ethanol followed by cold tap water. For antigen retrieval, a slide container was filled with antigen retrieval buffer (Tris-EDTA, pH 9.0), microwaved for 1 minute at a medium/high power level, then medium power level for 5 minutes, and cooled for 30 seconds, alternating 3 times. The container was cooled at room temperature, and then washed with TBS. The slides were blocked for 1 hour in TBS/0.01% Triton X-100/10% goat serum at room temperature. One hundred microliters of antibody was added to each slide. The slides were incubated at 4°C overnight, and then rinsed 3 times in TBS/0.025% Triton X-100. One hundred microliters of secondary antibody was added to each slide and slides were incubated at room temperature in the dark for 1 hour. Slides were rinsed twice in TBS/0.025% Triton X-100 and once in TBS. ProLong with DAPI (Invitrogen, P36931) was added to the slide and mounted with a coverslip. The slides were imaged using a Zeiss 710 confocal microscope. Staining of tissue was performed using the following antibodies and dilutions: anti-CK19 antibody (Abcam, ab52625; 1:400), anti-MUC2 antibody (BD, 555926; 1:250), anti-CDH1 (BD, 610181; 1:100), anti-synaptophysin (Abcam, ab32127; 1:250), anti-chromogranin (Abcam, ab15160; 1:100), and anti-SOX9 (EMD, AB5535; 1:500). Secondary antibodies were goat anti–rabbit IgG (H+L), Alexa Fluor 594 (Thermo Fisher Scientific, A-11037; 1:500) and goat anti–mouse IgG (H+L), Alexa Fluor 594 (Thermo Fisher Scientific, A-11005; 1:500).

For the H&E staining of PDOs, slides were deparaffinized and immersed in Gill’s no. 1 hematoxylin stain 3 times, rinsed with deionized water, dipped in 0.3% acid alcohol twice, rinsed with deionized water, dipped in aqueous ammonia, washed in tap water, dipped in 80% alcohol, and then counterstained with eosin Y with phloxine. The slides were dehydrated in 95% alcohol twice, 100% alcohol twice, and then xylene twice and a coverslip was applied. Slides of primary tumors were prepared for H&E staining and immunohistochemistry of selected surface proteins using standard clinical procedures through the Pathology Research Core in the Department of Pathology and Laboratory Medicine (Mayo Clinic). H&E stains for the primary tumor and corresponding PDO were graded by a pathologist in the Division of Anatomic Pathology, Mayo Clinic.

### Categorization of patient clinical response.

Patients were treated following sample collection based on their previous treatment history, actionable mutations (if available), and performance status independent of our study. The treatment efficacy was evaluated by CT or MRI imaging regularly per clinical practice standard or clinical trial protocol. Radiographic response to therapy was categorized as PD, SD, or PR according to the RECIST version 1.1 (58). In the CT scans, the circled regions represent the lesion of interest. Tumor size was measured using the Line Measurement Tool in QReads (Mayo Clinic). The longest diameter of the target lesion is reported on the CT scan.

### Categorization of PDO response and correlation to donor clinical response.

For clinical correlation, the PDOs were treated with either the first cancer agent(s) the patient received after sample collection, or the last cancer agent(s) the patient received prior to sample collection, with one exception. In the case of HO20, the PDO was treated with the third drug combination the patient received following biopsy. If the drug(s) used for patient treatment was not commercially available, a comparable commercially available drug was used for testing PDO response. For 8 of the 11 cases, we compared response to the same therapeutic agent(s) between the donor PDO and additional PDOs of the same cancer type to determine sensitivity. For 1 case, HO106, response was compared to 7 CRC PDOs since we did not have additional small bowel PDOs for comparison. Drug response for PDOs tested with the same therapeutic agent(s) was categorized as sensitive, intermediate, or resistant based on each PDO’s normalized AUC values for each drug using the Jenks Natural Breaks algorithm by BAMMtools, an R package (59), similarly to other studies (20, 51, 60). For 2 cases (HO17 and HO42), we did not have enough CCA PDOs available for comparison, so response was determined relative to published IC_50_ data for the same cancer type and drug. IC_50_ was determined using GraphPad Prism. Agreement between the patient tumor response and PDO response was categorized as match or mismatch using the following criteria: If donor tumor was PR, then the PDO response must be sensitive to be a match (intermediate or resistant is a mismatch). If donor tumor was SD, then PDO response must be categorized as sensitive or intermediate to be a match (resistant is a mismatch). If donor tumor is PD, then PDO response must be categorized as intermediate or resistant to be a match (sensitive is a mismatch). If multiple drugs were evaluated, the final determination on agreement was based on whether the PDO was sensitive to any of the drugs tested.

### Data availability.

The organoid RNA-seq data have been deposited in the NCBI Gene Expression Omnibus archive (GEO GSE212014).

### Statistics.

All drug response assays were performed with 3 or more biological replicates. *P* values indicate level of significance using Wilcoxon’s rank sum test between groups unless otherwise specified. A *P* value of less than 0.05 was considered significant. For all line graphs, the values reported equal the means of a minimum of 3 biological replicates expressed relative to the control as percentage cell viability. Bars in line graphs indicate ± SEM between biological replicates. For AUC, values were derived from the neighboring line graphs. Each dot represents 1 biological replicate. The line through the AUC equals the ± SD between the average AUCs for the biological replicates. The dot in the SD bar represents the mean AUC.

### Study approval.

Tissue samples from 163 patients enrolled in one of several prospective studies approved for tumor specimen collection at Mayo Clinic were used for the preparation of PDOs. Patient specimens included surgical tumor resections, EUS-FNB of the primary tumor, or US-biopsy of tumor metastases. These studies were approved by Mayo Clinic’s Institutional Review Board (IRB) under protocols 19-002657, 14-009985, 18-001386, 17-003174, and 66-06. Five pancreatic cancer specimens were obtained from Sanford Health in Sioux Falls, South Dakota, under their IRB protocol STUDY00001481. Research biopsies were obtained after the malignant lesion(s) were located and biopsied as part of routine clinical care. Tumor samples were sent for Tempus Targeted Sequencing as part of routine clinical care when possible. Following specimen collection, the laboratory assigned a lab number to the specimen and the following information was collected: patient name, clinic number, IRB number, diagnosis, tumor sample site, type of sample, and organoid initiation date. Patient treatment history was obtained when available. Seven PDXs used to generate additional pancreatic cancer PDOs were obtained from the Mayo Clinic Xenograft Program under IRB 66-06. These samples included HO162 (PAX238), HO163 (PAX4), HO219 (PAX265), HO222 (PAX139), HO227 (PAX297), HO228 (PAX300), and HO231 (PAX295). An additional 3 PDAC PDXs, HO159 (PDAC071T), HO160 (PDAC020T), and HO161 (PDAC009T), were provided by Nelson Dusetti and Juan L. Iovanna (CRCM, Marseille, France) to generate PDOs. Tissue transfer from the CRCM lab was approved under IRB protocol 2011-A01439-32.

## Author contributions

TLH conducted experiments, performed overall experimental design, optimized relevant protocols, and wrote the manuscript with MDT, HX, DLM, and MFZ. WJP assisted with experiment design, protocol optimization, organoid proliferation, and assisted with drug studies and genetic analysis of the PDOs. IPH and LLA optimized organoid proliferation protocols and assisted with drug studies in the organoids. DJK assisted with drug studies in the organoids. HX, JB, JHB, AM, JMH, TJSH, GMP, MJL, MZ, and WWM prepared the clinical protocols and coordinated obtaining patient samples. HX, JJL, RMC, and WWM extracted and reviewed patient clinical information. WJP, IPH, ANKK, EJT, ANS, MT, and TLH obtained patient specimens. RPG performed histological analysis of donor tumors and PDOs. MDT, TLH, CZ, and HL assisted in the bioinformatics analysis of the organoids. MTB assisted in genetic and CNV analysis of the organoids. MJT, ND, and JLI provided PDX tumors for the study and critically read the manuscript. BRK and SD assisted with genetic sequencing and analysis of the organoids. RLOO assisted with the illustrations in the manuscript. ALO critically read the manuscript and reviewed all statistical analyses. BRK and JDD provided reagents for genetic and transcriptomic analysis of the organoids. DDB, AAA, TSBS, and WWM provided conceptual framework for the project and critically read the manuscript.

## Supplementary Material

Supplemental data

ICMJE disclosure forms

Supplemental table 4

Supplemental table 5

## Figures and Tables

**Table 1:**
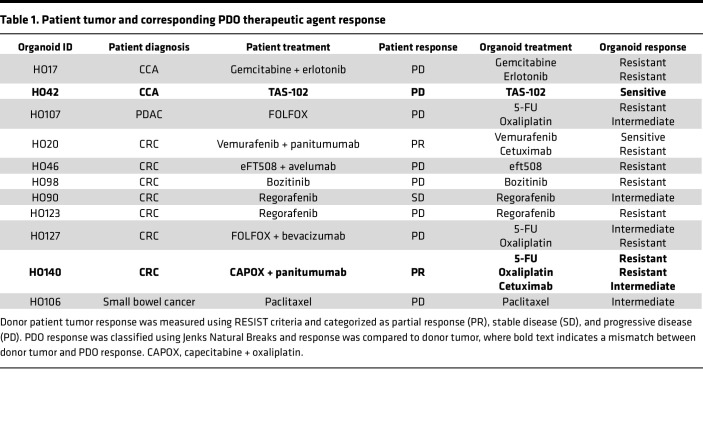
Patient tumor and corresponding PDO therapeutic agent response

## Version 1 10/18/2022

In-Press Preview

## Version 2 11/22/2022

Electronic publication
