# Supplementary material for: Culture media composition influences patient-derived organoid ability to predict therapeutic responses in gastrointestinal cancers
Source: JCI Insight. 2022 Nov 22;7(22):e158060. doi: 10.1172/jci.insight.158060 (PMC9746806; doi:10.1172/jci.insight.158060)
Supplement: Supplemental data [file jciinsight-7-158060-s064.pdf]

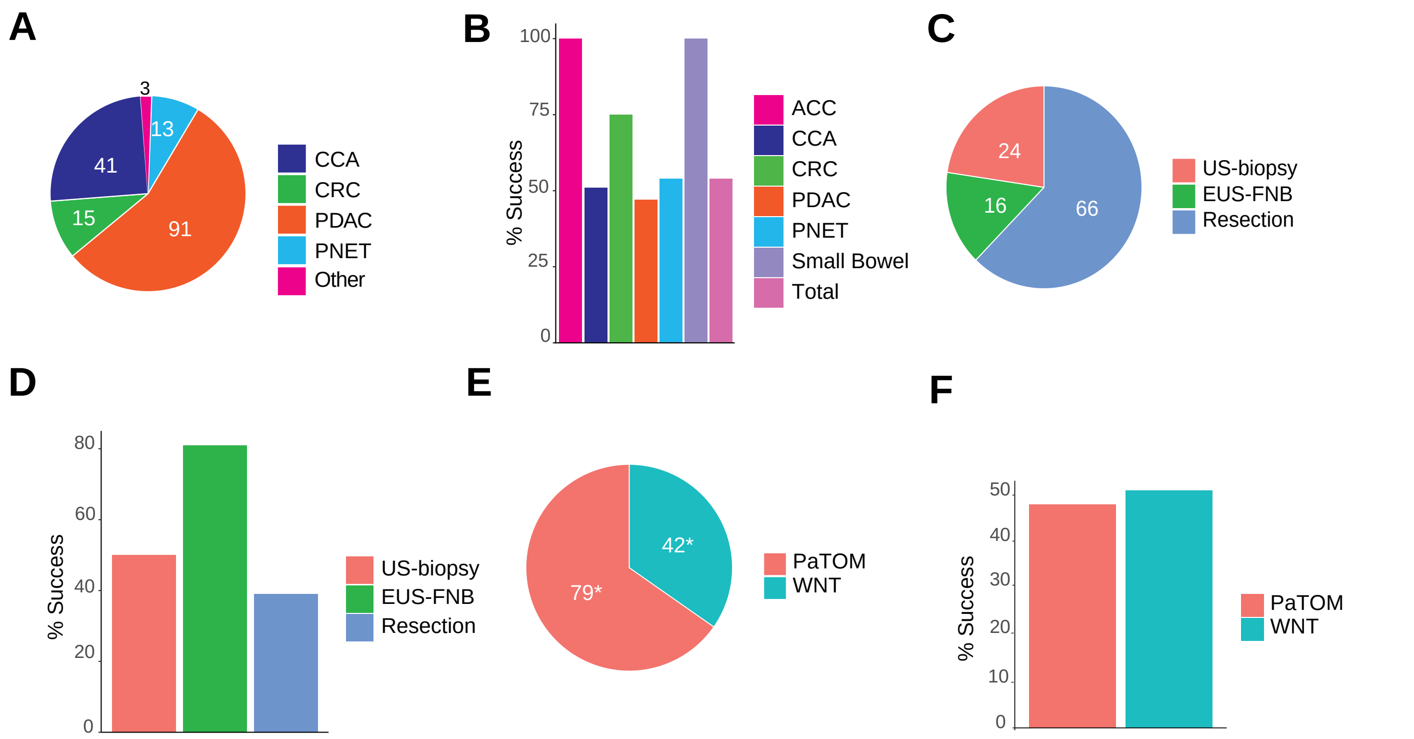

Supplemental Figure 1. Overview of specimen type and success rates for PDO formation from GI cancers. (A) Pie chart indicating number of PDOs attempted for each GI cancer type. Other = 2 ACC, 1 small bowel cancer; N = 163. (B) Bar graph indicating success rates for PDO formation per GI cancer type. (C) Pie chart indicating the number of pancreatic cancer specimens (ACC, PDAC, PNET) derived from US-biopsy, EUS-FNB, or surgical resection; N = 106. (D) Bar graph indicating the success rates for PDO formation by specimen type for pancreatic cancer. (E) Pie chart indicating the number pancreatic cancer PDOs grown in either PaTOM or WNT media; N = 121. *Fifteen organoids grown in both media. (F) Bar graph indicating the success rate for pancreatic cancer PDO formation by media type.

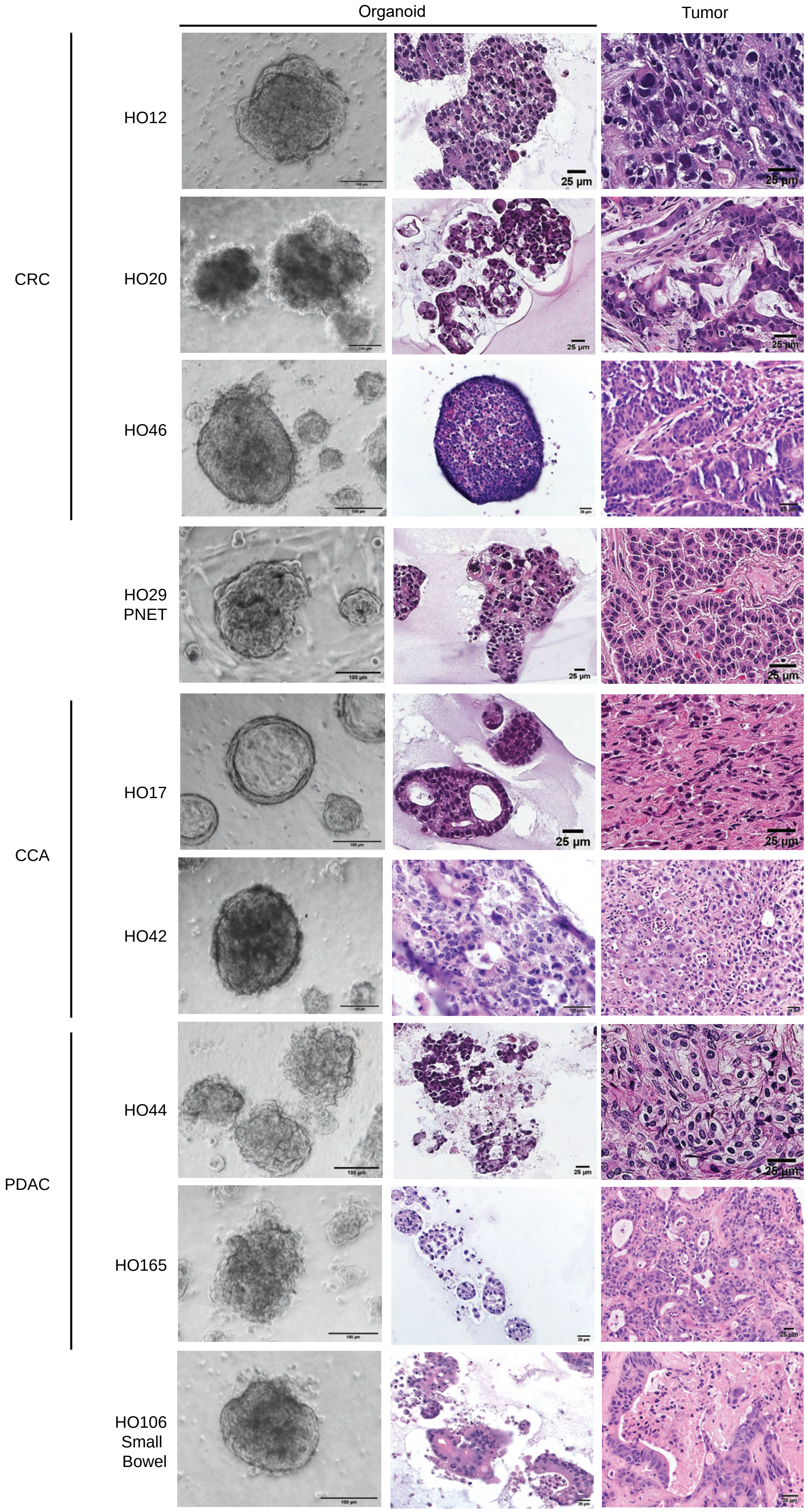

Supplemental Figure 2. Histological and morphologic characterization of PDOs. Organoid panels: Brightfield microscopy (scale bar = 100 µm) and H&E stains (scale bar = 25 µm). Tumor panels: H&E stains of corresponding donor tumor (scale bar = 25 µm); N = 9. Cancer type and PDO number shown next to the images.

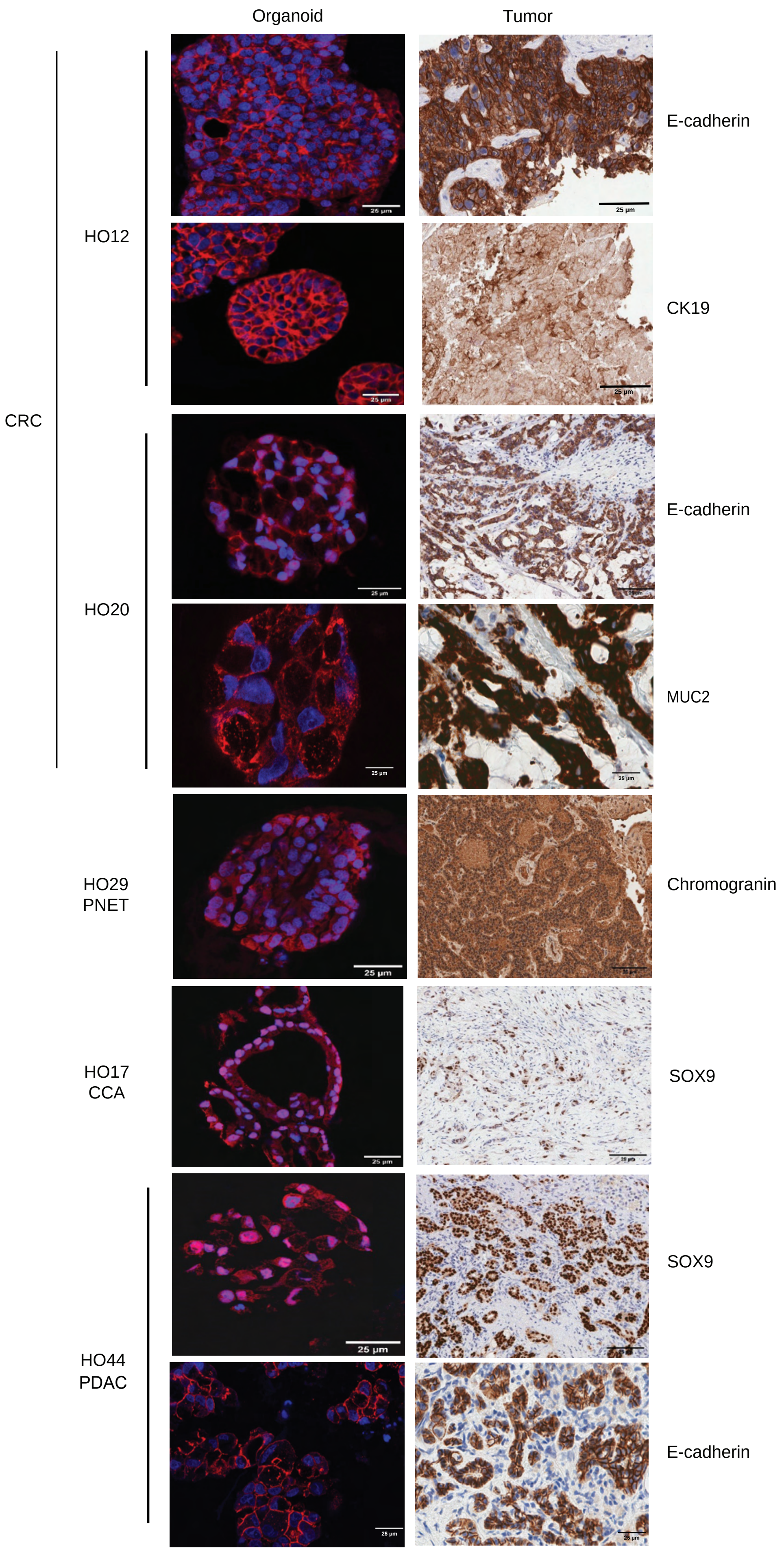

Supplemental Figure 3. Immunohistological characterization of PDOs. Organoid panels: Confocal immunofluorescence images of PDOs stained for common protein markers for each cancer type (scale bar = 25 μm). E-cadherin, CK19, MUC2, Chromogranin, and SOX9 shown in red and DAPI in blue. Tumor panels: Immunohistochemistry of corresponding donor tumors (scale bar = 25 μm); N = 5. Cancer type and PDO number shown next to the images.

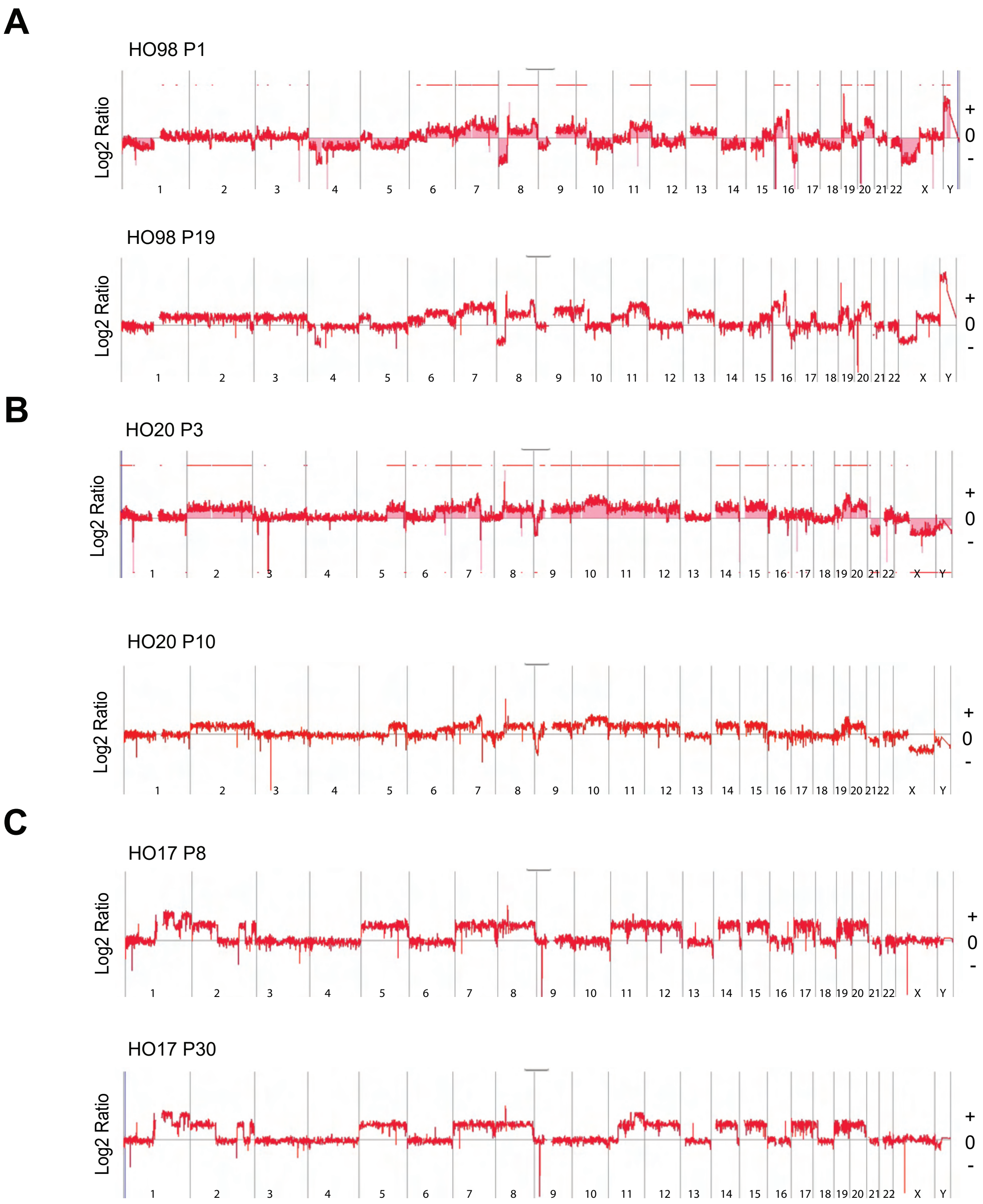

Supplemental Figure 4. CNV Profiles of PDOs demonstrate chromosomal stability across multiple passages. (A) CRC PDO HO98 CNV Profile at Passage 1 and Passage 19. (B) CRC PDO HO20 CNV Profile at Passage 3 and Passage 10. (C) CCA PDO HO17 CNV Profile at Passage 8 and Passage 30. The x axis represents chromosome number and position and the y axis shows log2 ratios for each chromosome.

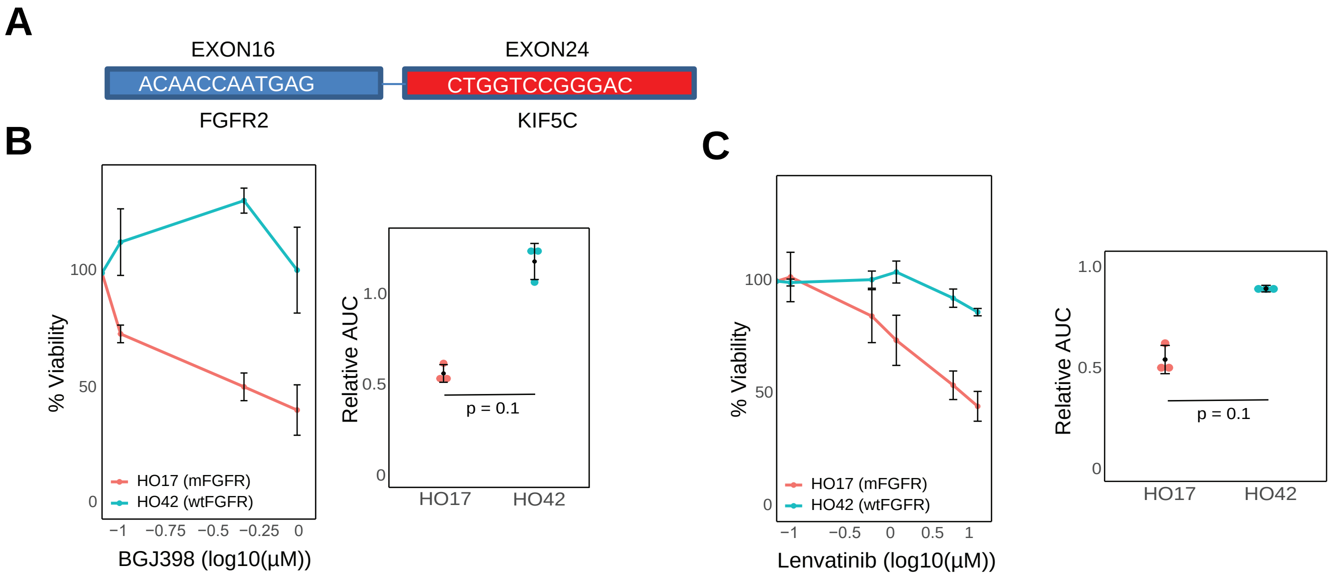

D

Organoid	Media	KRAS Status	FGFR Status	Pathogenic Variant
HO44	PaTOM	Wild Type	Wild Type	NA
HO107	PaTOM	c.35G>A	Wild Type	NA
HO219	PaTOM	c.35G>T	Mutant	FGFR1-ERLIN2 Fusion

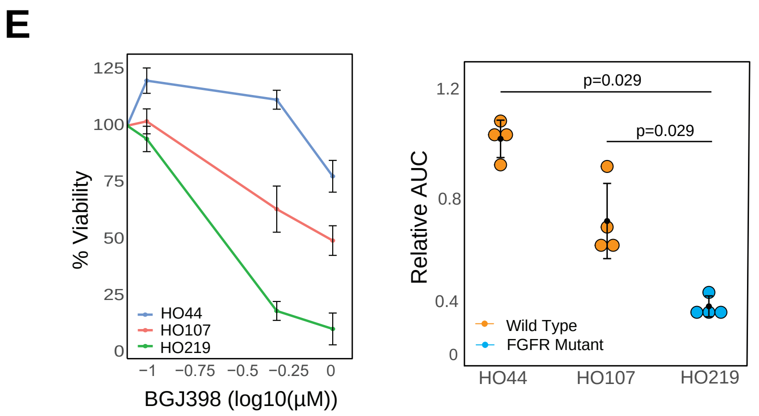

F

Organoid	Media	KRAS Status	HRD Status	Pathogenic Variant
HO162	PaTOM	c.35G>A	HRD	BRCA2 c.1307dupA
HO44	PaTOM	Wild Type	Non-HRD	NA
HO107	PaTOM	c.35G>A	Non-HRD	NA

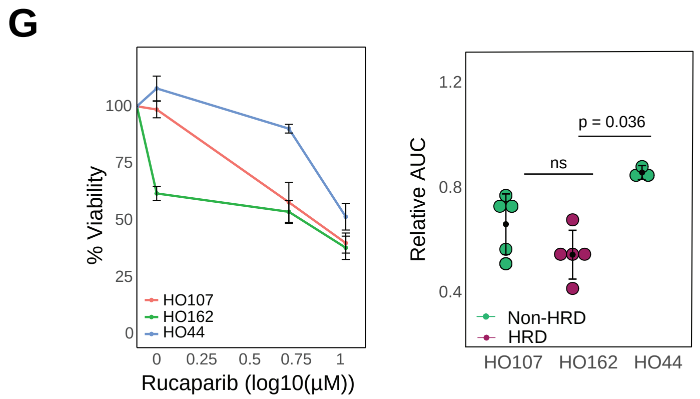

Supplemental Figure 5. Mutational status in PDOs associated with sensitivity to cancer therapeutics. (A) CCA PDO HO17 and donor tumor contains a FGFR2-KIF5C fusion. (B, C) Percent cell viability and relative AUC values in response to (B) BGJ398 and (C) Lenvatinib treatment for CCA PDO HO17 and wild type FGFR CCA PDO HO42; N = 2. (D) KRAS and FGFR mutation status of 3 PDAC PDOs grown in PaTOM media. (E) PDAC PDO percent cell viability and relative AUC values in response to BGJ398 in PaTOM media; N = 3. (F) KRAS and HRD mutation status of 3 PDAC PDOs in PaTOM media. (G) Percent cell viability and relative AUC values of PDAC PDOs in response to Rucaparib in PaTOM media; N = 3. p values indicate level of significance using Wilcoxon Rank Sum test between mutant and wild type PDOs.

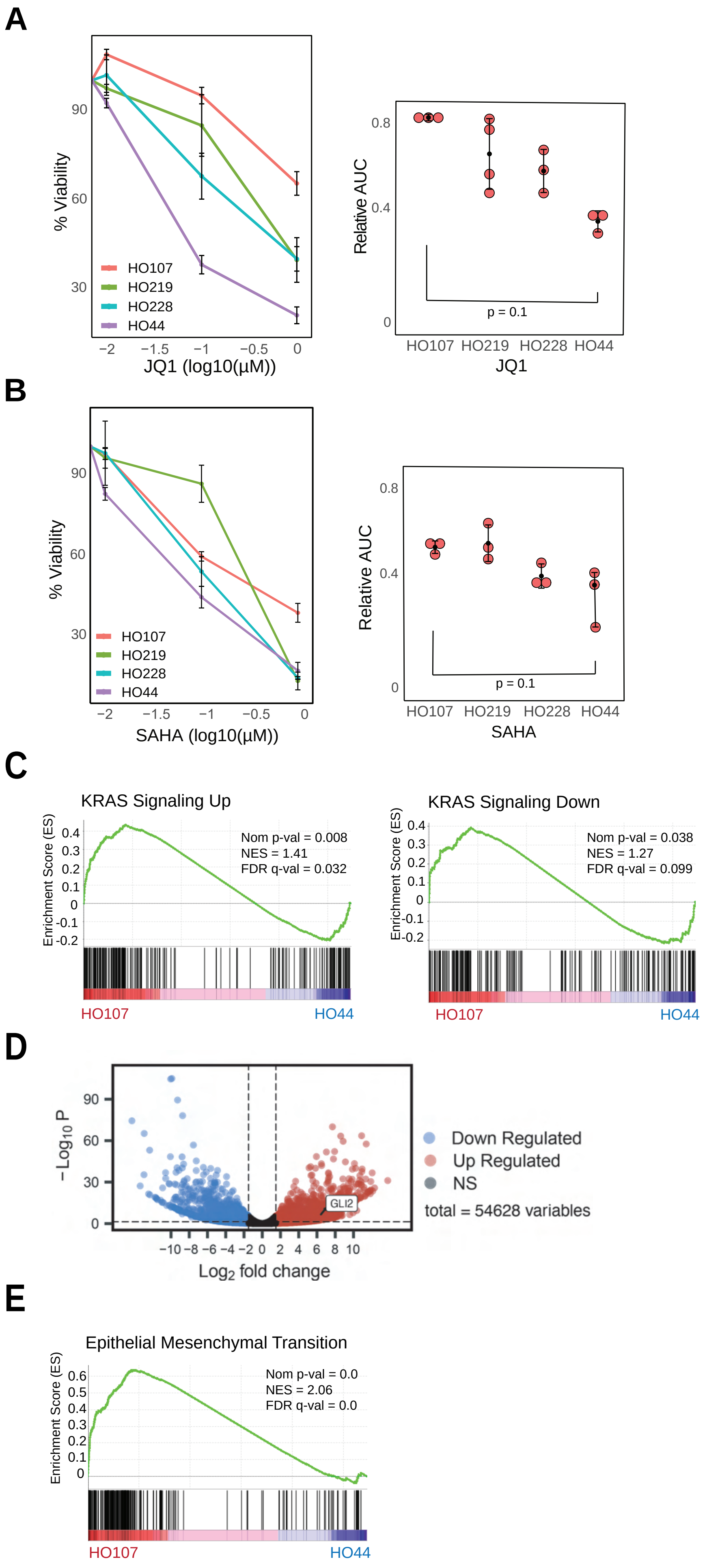

Supplemental Figure 6. Pancreatic cancer PDOs transcriptomic signatures and drug response to epigenetic therapies. (A) Percent cell viability and relative AUC values for PDAC PDOs in response to JQ1 in PaTOM media; N = 4. (B) Percent cell viability and relative AUC values for PDAC PDOs in response to SAHA in PaTOM media; N = 4. p values indicate levels of significance using Wilcoxon Rank Sum test between the AUCs of the most sensitive (HO44) and resistant (HO107) PDO. (C) Gene sets enriched in PDAC PDO HO107 using GSEA for Hallmark gene sets. (D) Volcano plot showing genes down-regulated and up-regulated in HO107 where base mean ≥1, Padj ≤0.05, and |FC| >2. (E) Gene set enriched in HO107 using GSEA for Hallmark gene sets.

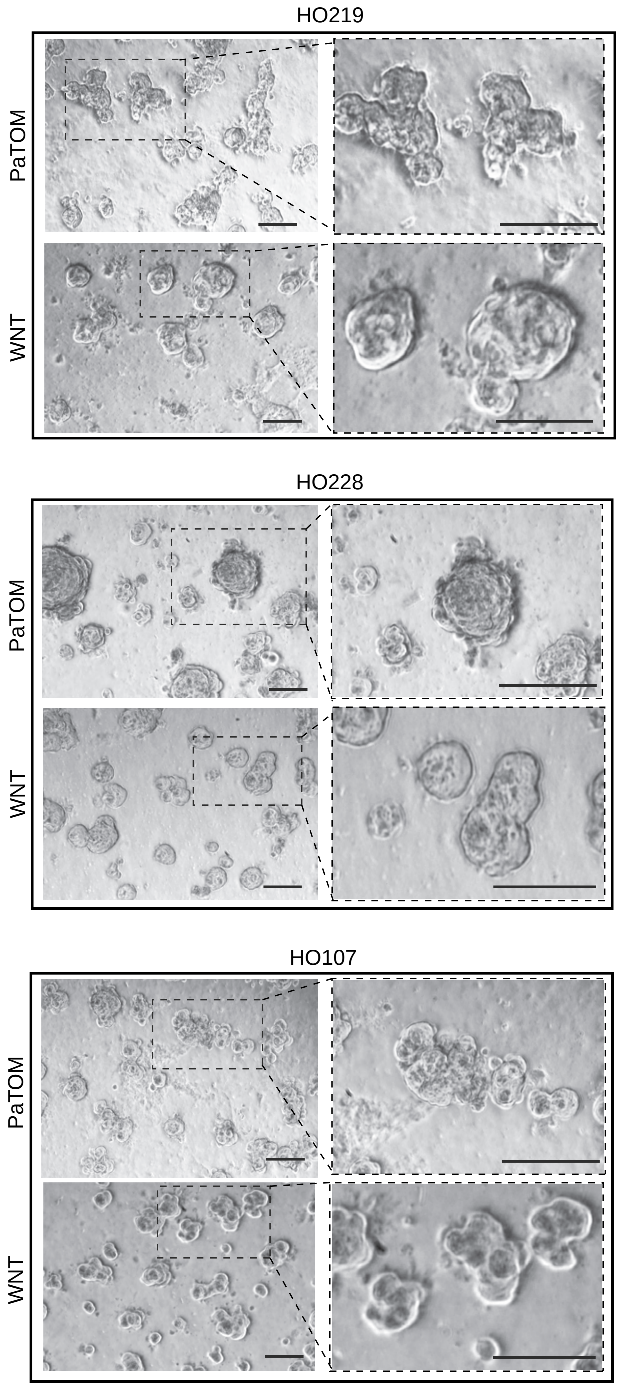

Supplemental Figure 7. Morphology of PDAC PDOs in WNT and PaTOM media. Brightfield imaging of PDAC PDOs grown in WNT and PaTOM media at two magnifications (scale bars = 100 µm); N = 3.

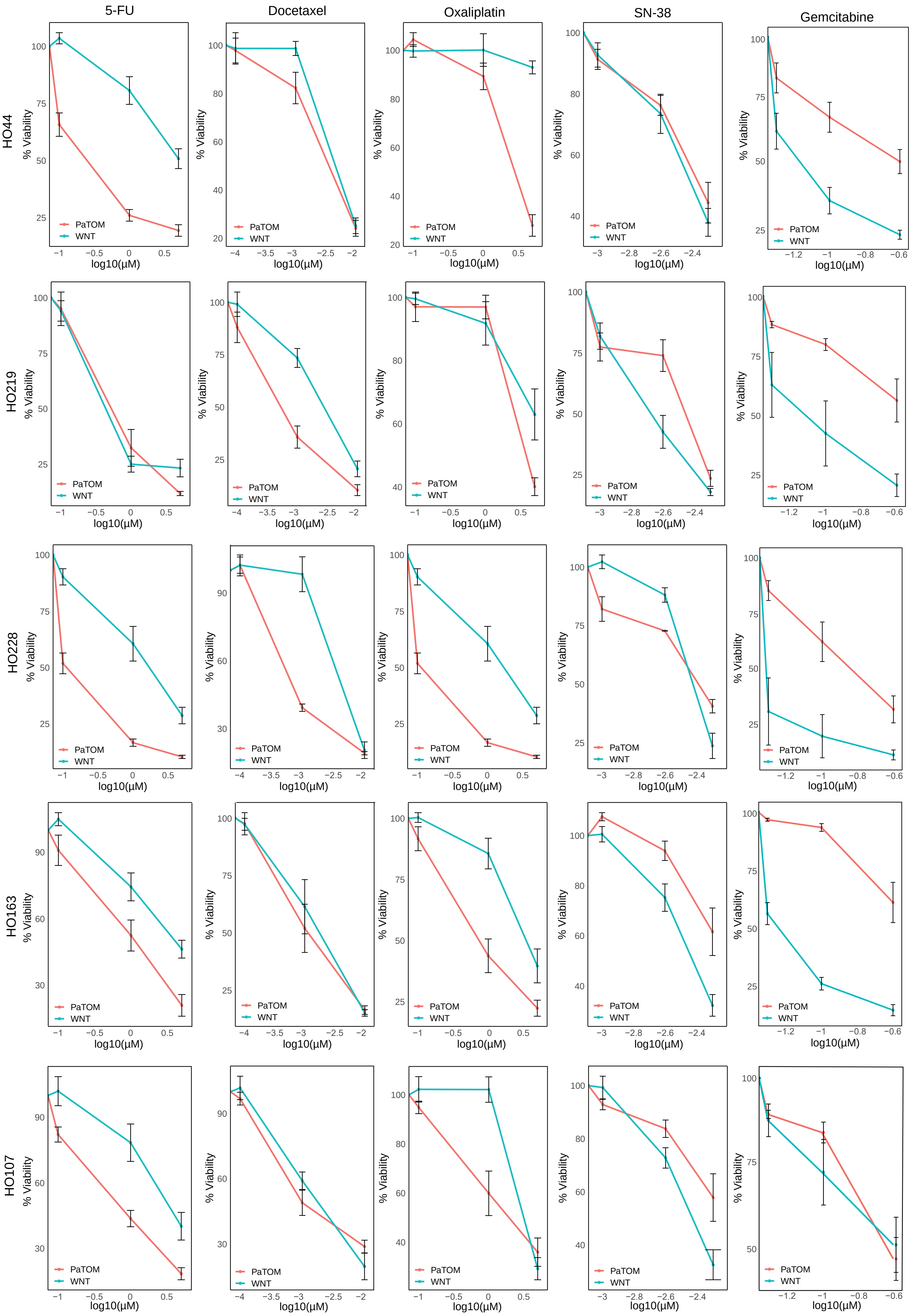

5-FU
Docetaxel
Oxaliplatin
SN-38
Gemcitabine
HO44
HO219
HO228
HO163
HO107
% Viability
log10(μM)
PaTOM
WNT

Supplemental Figure 8. PDAC PDO drug response to chemotherapies in PaTOM and WNT media. Percent cell viability for PDAC PDOs grown in PaTOM (red) and WNT (blue) media after treatment with chemotherapy including 5-FU, Docetaxel, Oxaliplatin, SN-38, and Gemcitabine; N = 5.

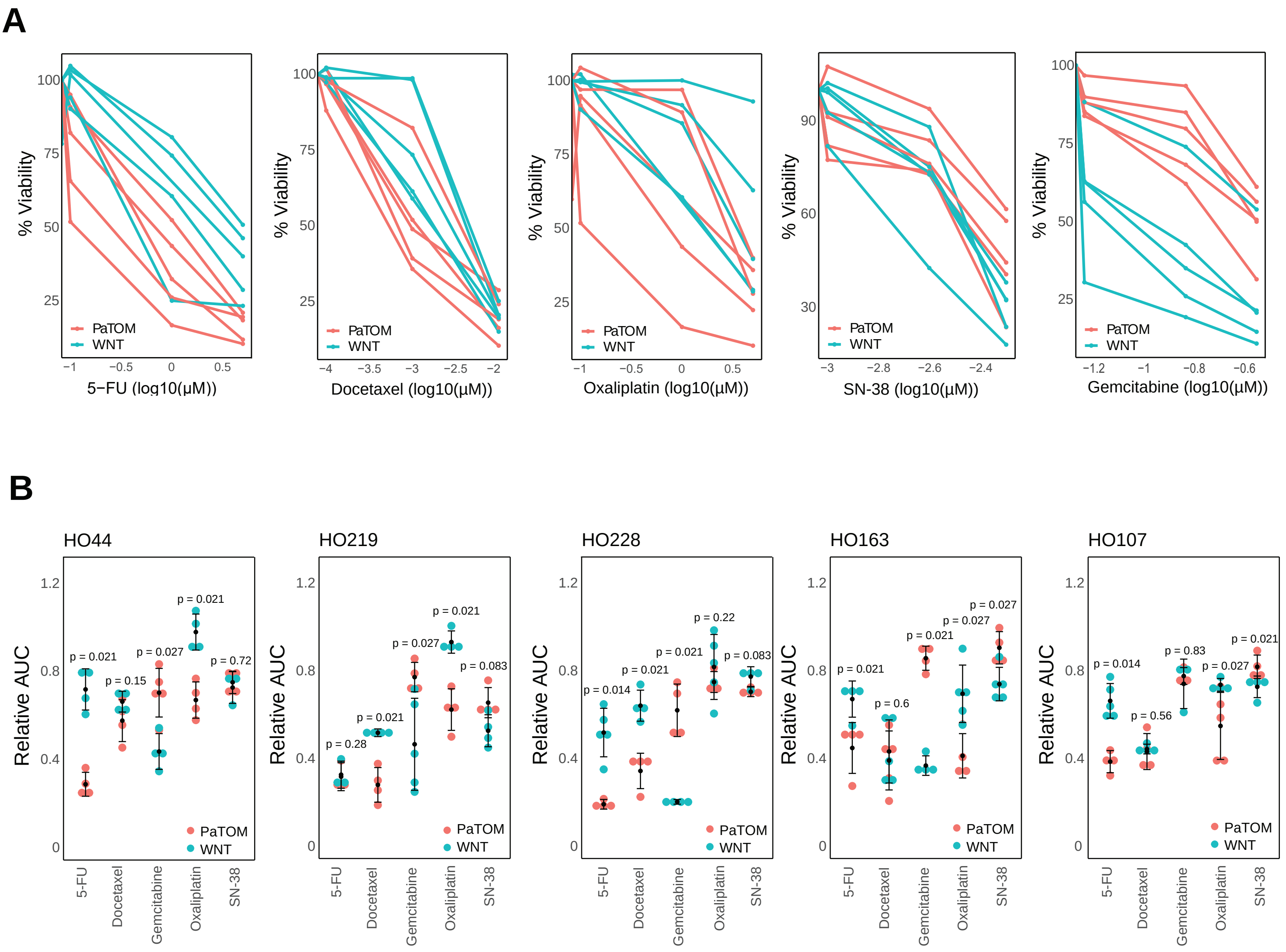

Supplemental Figure 9. Summary of PDAC PDOs drug response to chemotherapies in PaTOM and WNT media. (A) Comparison of percent cell viability for PDAC PDOs grown in PaTOM (red) and WNT (blue) media after treatment with chemotherapy including 5-FU, Docetaxel, Oxaliplatin, SN-38, and Gemcitabine; N = 10. (B) Summary of relative AUC for each PDAC PDO in WNT and PaTOM to 5-FU, Docetaxel, Oxaliplatin, SN-38, and Gemcitabine; N = 5. Each dot represents the relative AUC value for one replicate for each PDAC PDO derived from the viability line graphs in Supplementary Figure 8. p values indicate level of significance using Wilcoxon Rank Sum test between AUC of PDOs grown in PaTOM and WNT media.

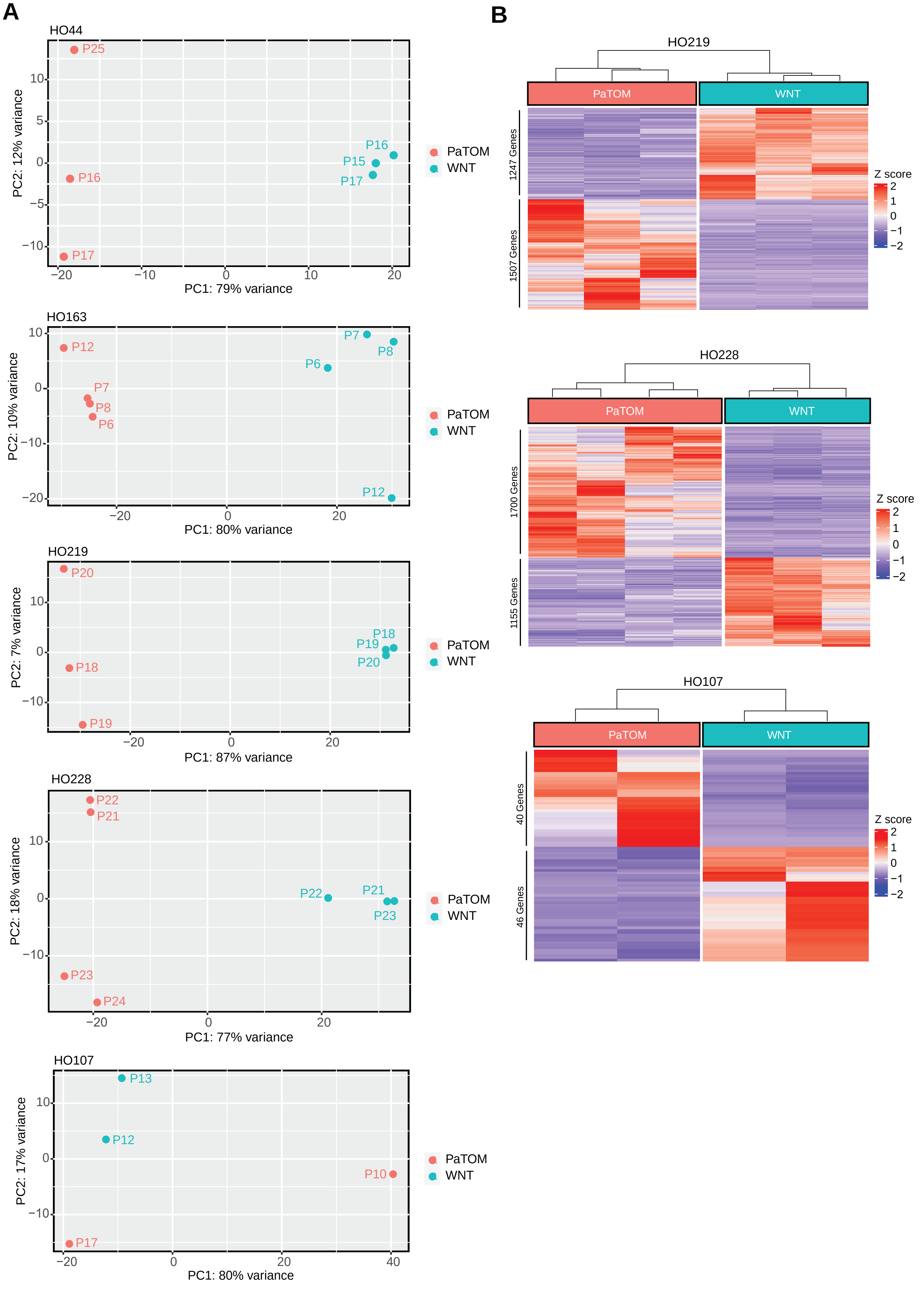
A
B
HO44
P25
P16
P17
P16
P15
P17
PC2: 12% variance
PC1: 79% variance
PaTOM
WNT
HO163
P12
P7
P8
P6
P7
P8
P6
P12
PC2: 10% variance
PC1: 80% variance
HO219
P20
P18
P19
P18
P19
P20
PC2: 7% variance
PC1: 87% variance
HO228
P22
P21
P23
P24
P22
P21
P23
PC2: 18% variance
PC1: 77% variance
HO107
P13
P12
P10
P17
PC2: 17% variance
PC1: 80% variance
HO219
PaTOM
WNT
1247 Genes
1507 Genes
Z score
HO228
1700 Genes
1155 Genes
HO107
40 Genes
46 Genes

Supplemental Figure 10. PDAC PDOs grown in two culture media show distinct transcriptomic signatures. (A) PCA plot of PDAC PDOs in PaTOM and WNT media; N = 5. P=Passage number. (B) Heatmap showing clustering by differentially expressed genes for PDAC PDOs cultured in WNT and PaTOM media with a base mean ≥1, Padj ≤0.05, and |FC| >2; N = 3.

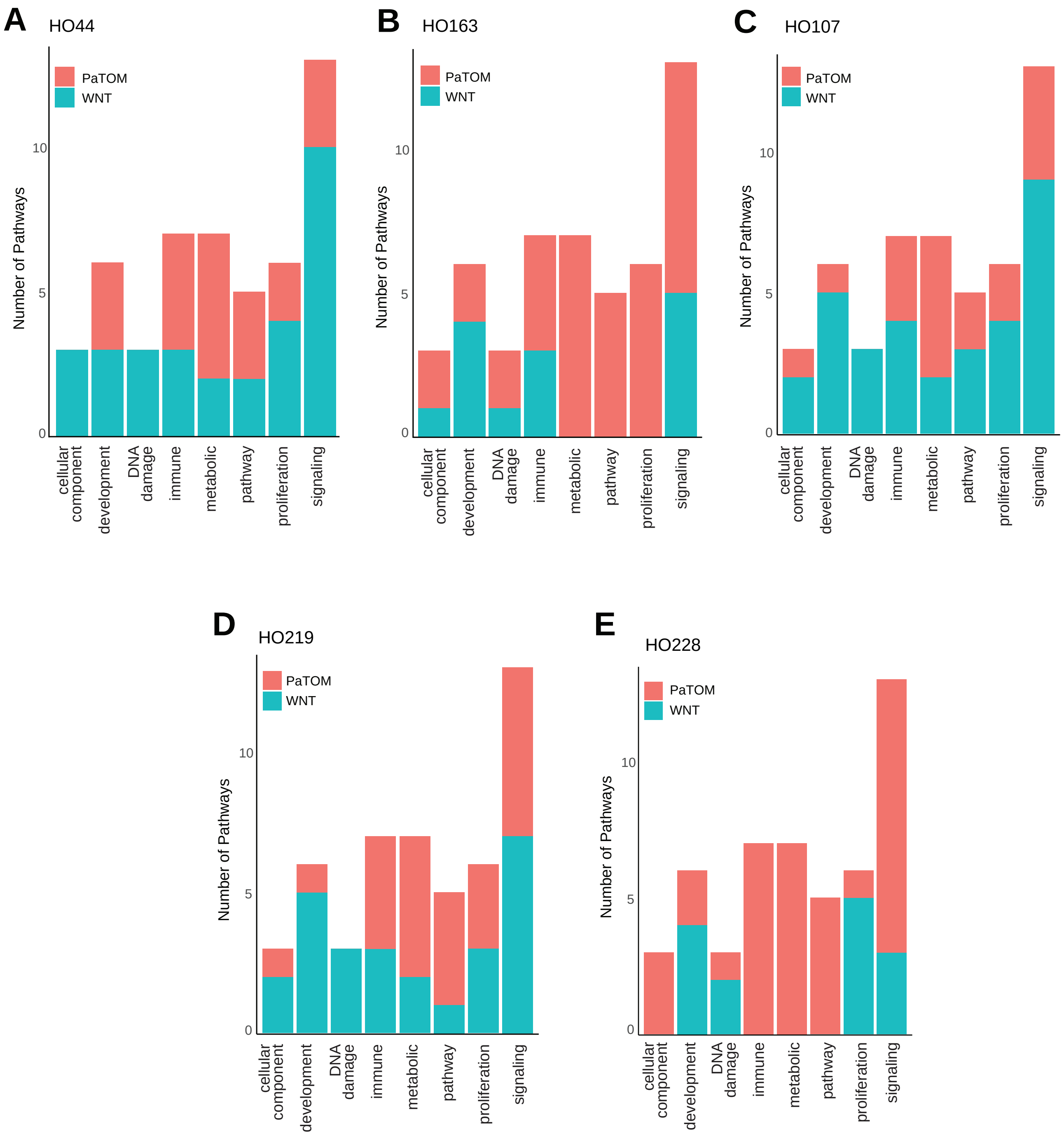

Supplemental Figure 11. PDAC PDOs grown in two culture media show enrichment of different processes. Summary of enrichment for each process category defined by GSEA Hallmarks gene sets using RNA-seq expression data for PDAC PDOs (A) HO44, (B) HO163, (C) HO107, (D) HO219, and (E) HO228 in PaTOM and WNT media. All gene sets reported as enriched by GSEA in Supplemental Table 5 were included regardless of p value.

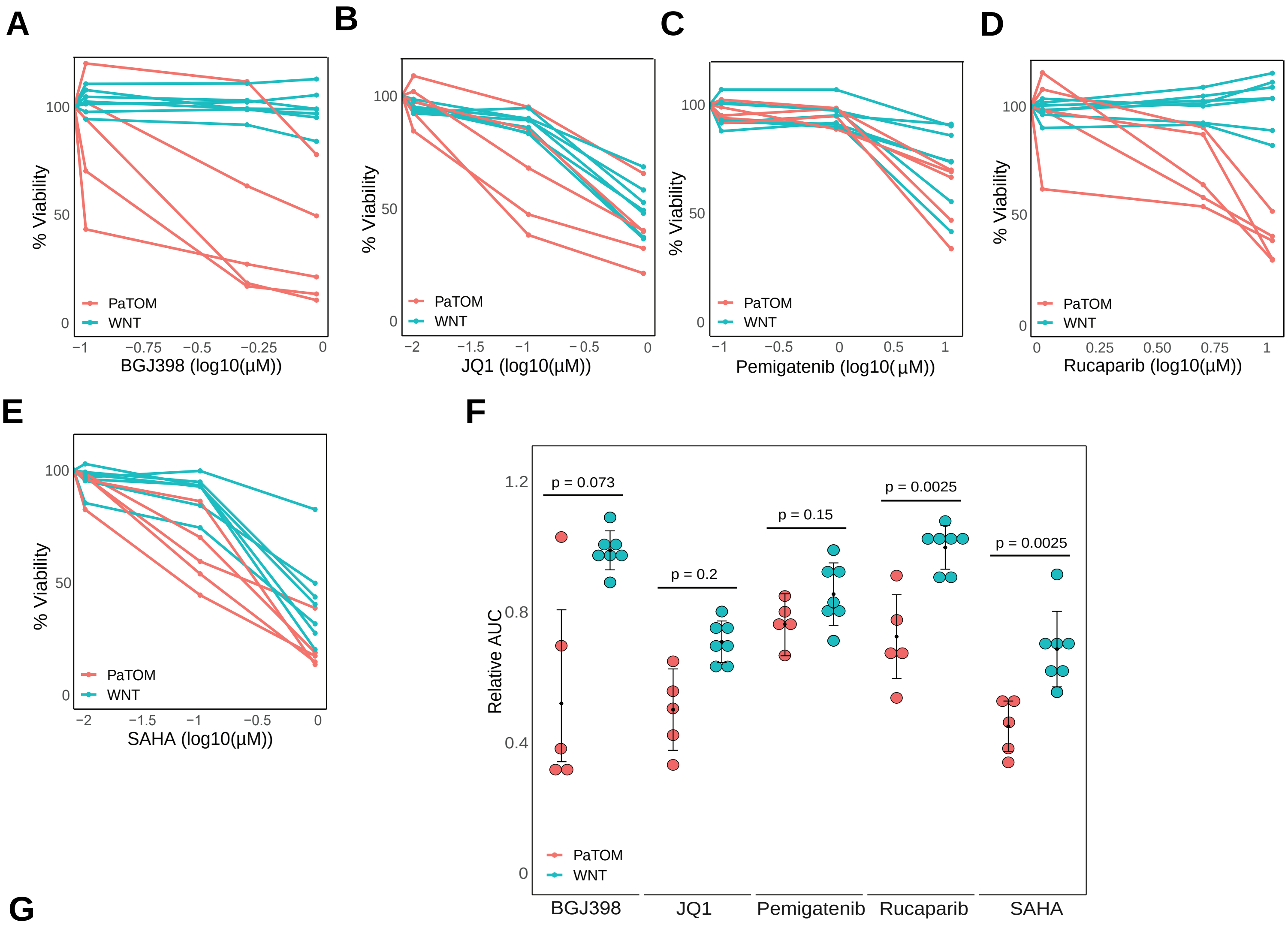

G

Organoid	Media	KRAS Status	HRD Status	Pathogenic Variant
HO133	WNT	Wild Type	HRD	BRCA1 c.1067A>G
HO159	WNT	c.35G>A	HRD	ATM c.5557G>A
HO161	WNT	c.34G>C	HRD	BRCA1 c.2722G>T; BRCA2 c.125A>G
HO165	WNT	c.183A>T	HRD	ATM c.3161C>G; ATM c.2572T>C
HO145	WNT	c.35G>T	Non-HRD	NA
HO153	WNT	c.35G>T	Non-HRD	NA
HO160	WNT	c.35G>A	Non-HRD	NA

H

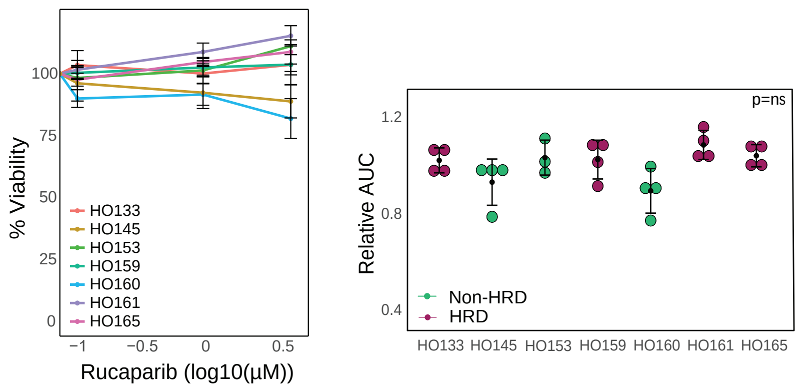

Supplemental Figure 12. PDAC PDOs grown in WNT media are associated with a more resistant phenotype to targeted therapies. (A-E) Percent cell viability for 5 PDAC PDOs grown in PaTOM (red) and 7 PDAC PDOs grown in WNT (blue) media after treatment with targeted therapy agents (A) BGJ398 (B) JQ1 (C) Pemigatenib (D) Rucaparib (E) SAHA; N = 12. (F) Relative AUC of PDAC PDOs in response to targeted therapies for 5 PDAC PDOs cultured in PaTOM and 7 PDOs cultured in WNT media; N = 12. Each dot represents the average relative AUC values for one PDAC PDO derived from the viability line graphs in A-E. p values indicate level of significance using Wilcoxon Rank Sum test between AUC of PDOs grown in PaTOM and WNT media. PaTOM BGJ398 and Rucaparib data extracted from Supplementary Figure 5. PaTOM JQ1 and SAHA data extracted from Supplementary Figure 6. (G) KRAS and HRD mutation status of PDAC PDOs in WNT media; N = 7. (H) Percent cell viability and relative AUC values of PDAC PDOs in response to Rucaparib in WNT media; N = 7. p values indicate level of significance using Kruskal-Wallis test between groups.

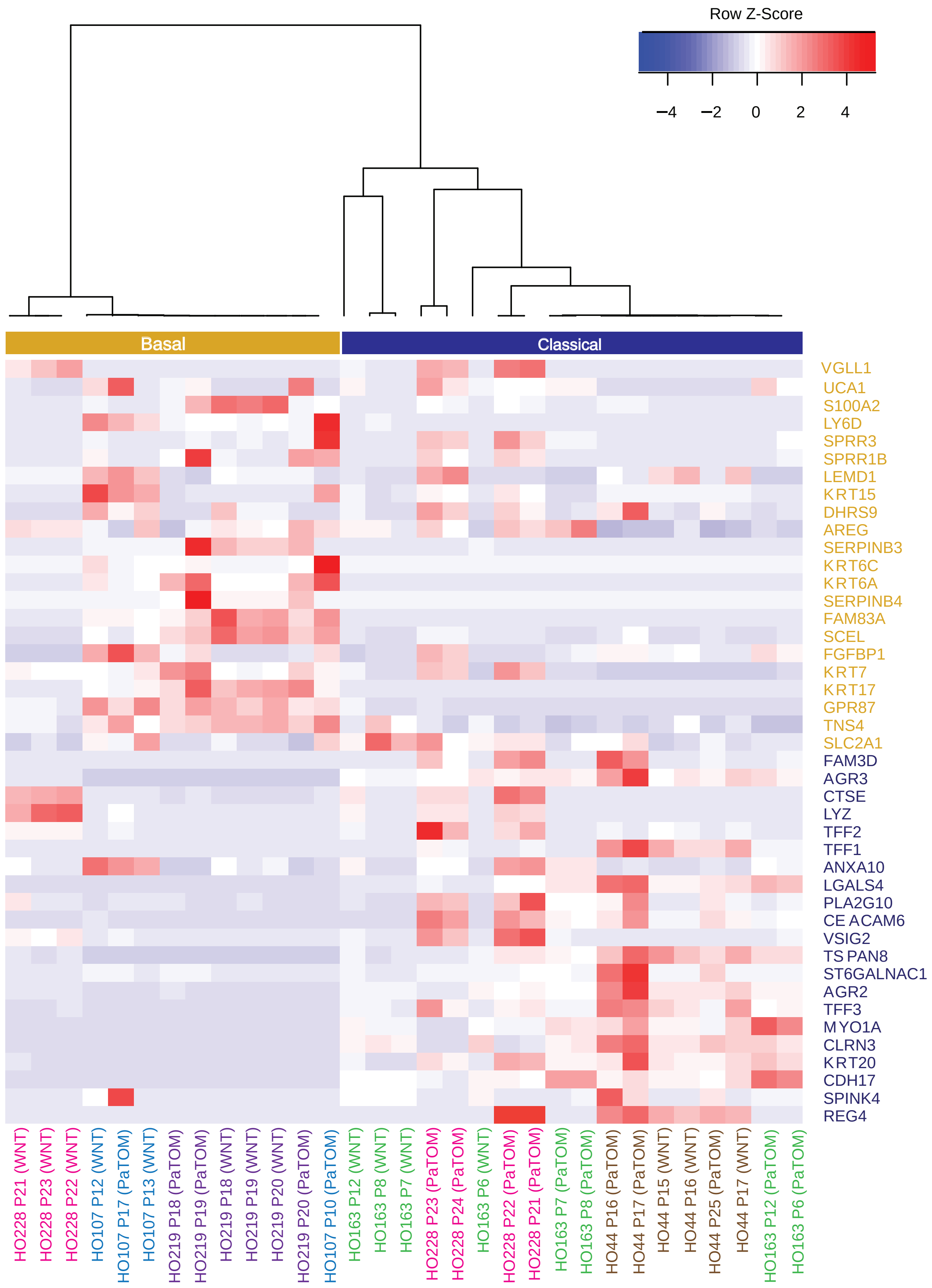

Supplemental Figure 13. Transcriptomic subtyping of pancreatic cancer PDOs. PDAC PDOs were categorized as classical or basal subtype by consensus clustering of RNA-seq z-scores for 5 PDAC PDOs in WNT and PaTOM media over multiple passages using the Moffit Gene Set. The genes in gold are associated with a basal gene signature and the genes in blue are associated with a classical gene signature. The organoid culture media in which each PDO was grown is listed in parenthesis. Each color represents all the samples associated with one PDO in PaTOM and WNT; N = 31. P = Passage number.

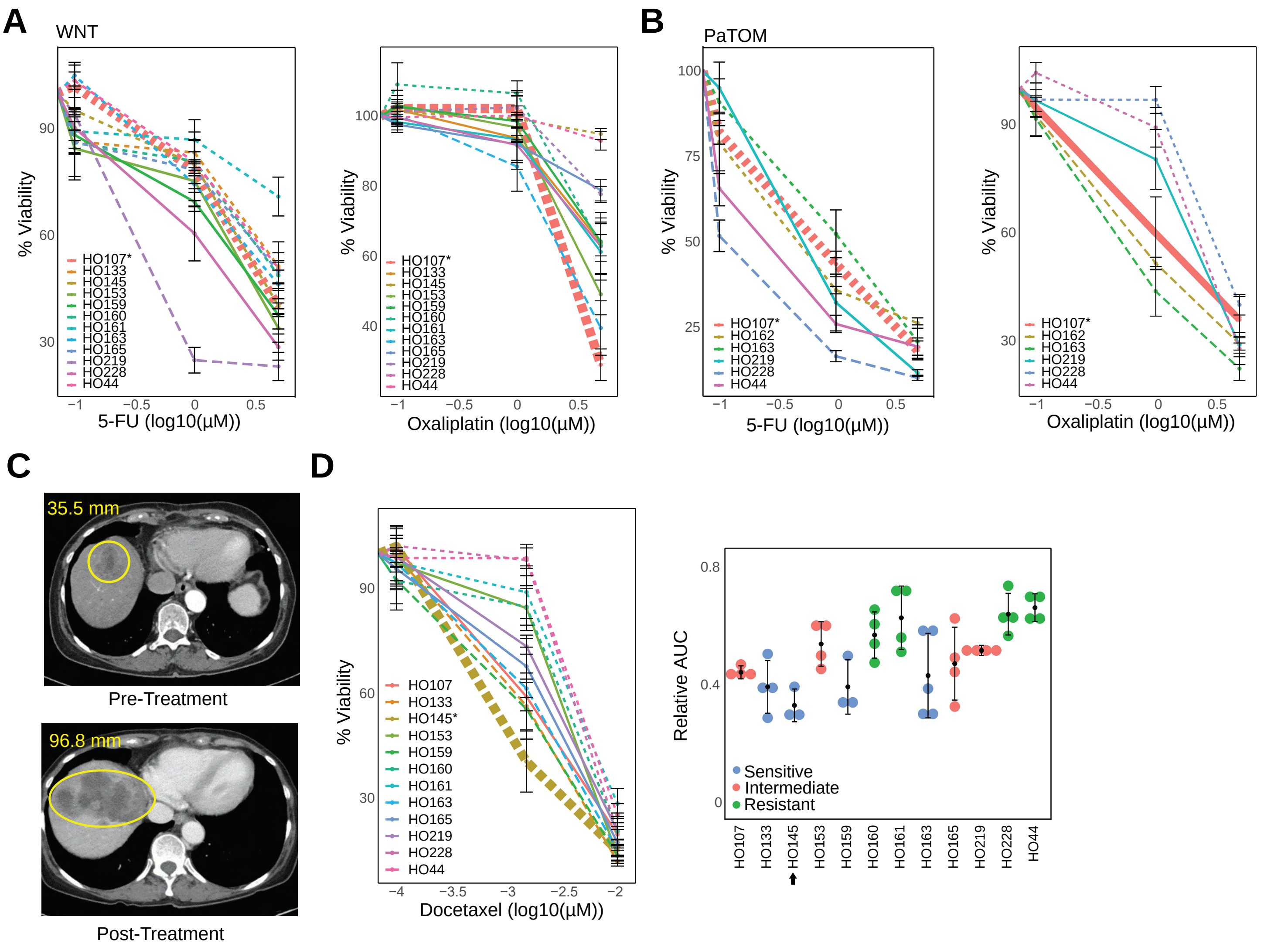

Supplemental Figure 14. The effect of PDO culture media on clinical correlation. (A) Percent cell viability for PDAC PDO HO107 and additional PDAC PDOs in response to 5-FU and Oxaliplatin in WNT; N = 12. (B) Percent cell viability for PDAC PDO HO107 and additional PDAC PDOs in response to 5-FU and Oxaliplatin in PaTOM; N = 6. (C) CT scans of donor patient for PDO HO145 pre- and post-treatment with PD showing the lesion increased from 35.5 mm to 96.8 mm. (D) Percent cell viability and relative AUC values in response to Docetaxel for PDAC PDO HO145 and additional PDAC PDOs grown in WNT media; N = 12. For line graphs, dashed lines indicate sensitive, solid lines indicate intermediate, dotted lines indicate resistant categorized using the Jenks Natural Breaks algorithm. The HO number with an asterisk and the thickest line in the line graphs indicates PDO response corresponding to the patient tumor response shown in the CT scan. The arrow next to the HO number in the relative AUC graph indicates the PDO corresponding to the donor tumor.

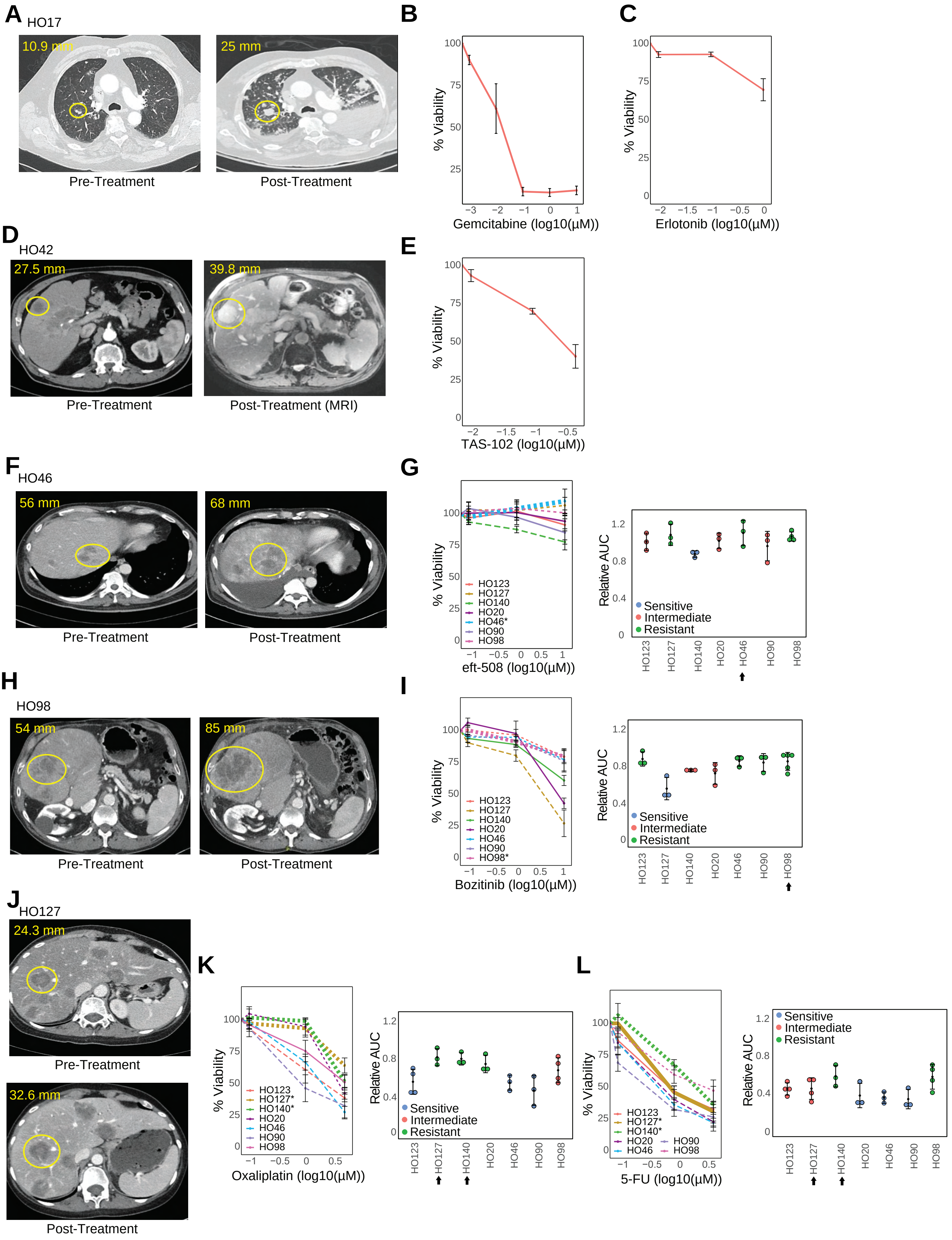

A HO17
10.9 mm
25 mm
Pre-Treatment
Post-Treatment
B
% Viability
Gemcitabine (log10(μM))
C
% Viability
Erlotonib (log10(μM))
D HO42
27.5 mm
39.8 mm
Pre-Treatment
Post-Treatment (MRI)
E
% Viability
TAS-102 (log10(μM))
F HO46
56 mm
68 mm
Pre-Treatment
Post-Treatment
G
% Viability
HO123
HO127
HO140
HO20
HO46*
HO90
HO98
eft-508 (log10(μM))
Relative AUC
Sensitive
Intermediate
Resistant
HO123 HO127 HO140 HO20 HO46 HO90 HO98
H
HO98
54 mm
85 mm
Pre-Treatment
Post-Treatment
I
% Viability
HO123
HO127
HO140
HO20
HO46
HO90
HO98*
Bozitinib (log10(μM))
Relative AUC
Sensitive
Intermediate
Resistant
HO123 HO127 HO140 HO20 HO46 HO90 HO98
J HO127
24.3 mm
Pre-Treatment
32.6 mm
Post-Treatment
K
% Viability
HO123
HO127*
HO140*
HO20
HO46
HO90
HO98
Oxaliplatin (log10(μM))
Relative AUC
Sensitive
Intermediate
Resistant
HO123 HO127 HO140 HO20 HO46 HO90 HO98
L
% Viability
HO123
HO127*
HO140*
HO20
HO46
HO90
HO98
5-FU (log10(μM))
Relative AUC
Sensitive
Intermediate
Resistant
HO123 HO127 HO140 HO20 HO46 HO90 HO98

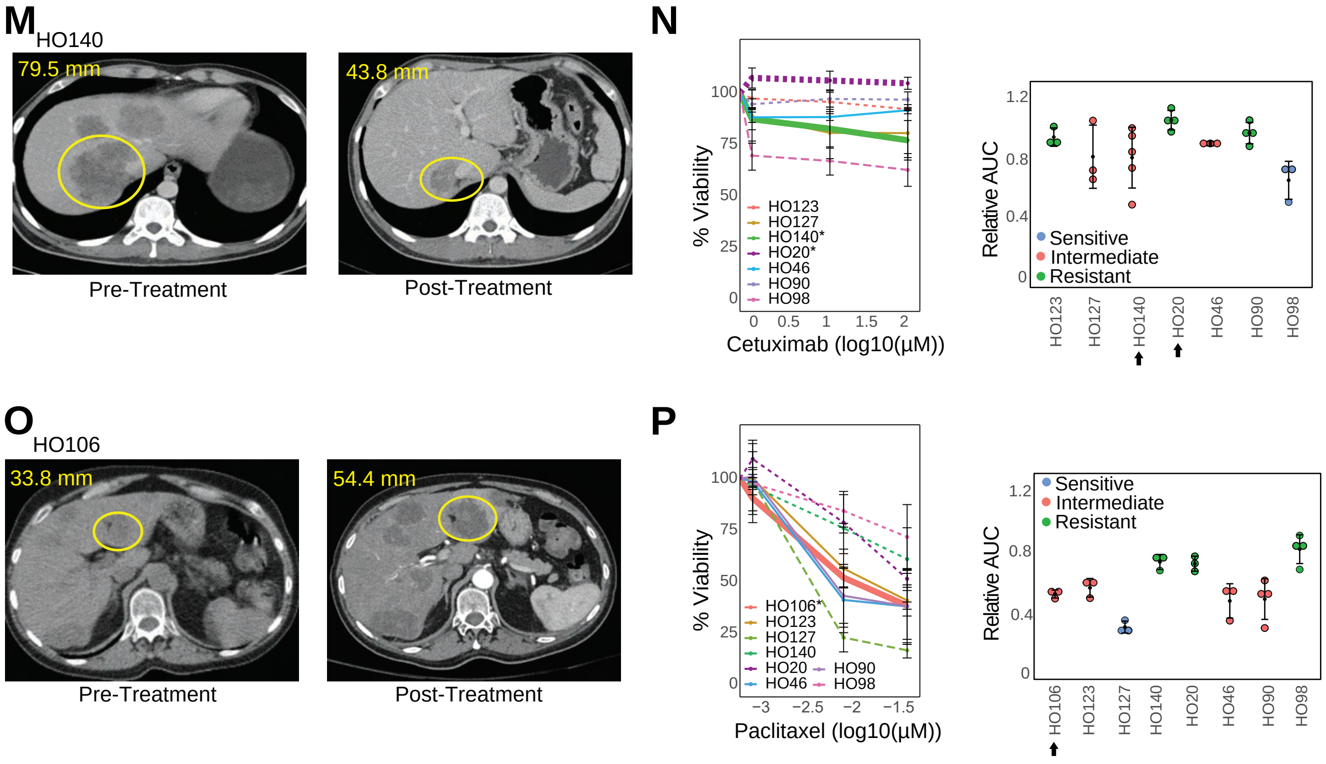

Supplemental Figure 15. Donor patient CT scans and drug responses of corresponding PDOs. (A) CT scans of donor patient pre- and post-treatment showing PD (HO17). (B, C) Percent cell viability for HO17 in response to (B) Gemcitabine and (C) Erlotinib. (D) Scans of donor patient pre-treatment (CT) and post-treatment (MRI) showing PD (HO42). (E) PDO HO42 percent cell viability in response to TAS-102. (F) CT scans of donor patient pre- and post-treatment showing PD (HO46). (G) Percent cell viability and relative AUC values in response to eft-508 for 7 CRC PDOs, including HO46. (H) CT scans of donor patient pre- and post-treatment showing PD (HO98). (I) Percent cell viability and relative AUC values in response to Bozitinib for 7 CRC PDOs, including HO98. (J) CT scans of donor patient pre- and post-treatment showing PD (HO127). (K, L) Percent cell viability and relative AUC values in response to (K) Oxaliplatin and (L) 5-FU for 7 CRC PDOs, including HO127. (M) CT scans of donor patient pre- and post-treatment showing PR (HO140). (N, K, L). Percent cell viability and relative AUC values in response to (K) Oxaliplatin, (L) 5-FU (L), and (N) Cetuximab for 7 CRC PDOs, including HO140. (O) CT scans of donor patient pre- and post-treatment showing PD (HO106). (P) Percent cell viability and relative AUC values in response to Paclitaxel for 8 PDOs, including HO106. For line graphs, dashed lines indicate sensitive, solid lines indicate intermediate, dotted lines indicate resistant PDOs categorized using Jenks Natural Breaks algorithm. The HO number with an asterisk and the thickest line in the line graphs indicates PDO response corresponding to the patient tumor response shown in the CT scan (some drug responses correspond to multiple patients). The arrow next to the HO number in the relative AUC graph indicates the PDO corresponding with the donor tumor.

Patient Data Table (n=163)	
**Age at Diagnosis**	
Mean	65
Range	24-84
**Sex**	
Female	73 (45%)
Male	90 (55%)
**Diagnosis**	
Cholangiocarcinoma	41 (25%)
Pancreatic Cancer	106 (65%)
Colorectal Cancer	15 (9%)
Small Bowel Cancer	1
**Sample Location**	
Liver	75 (46%)
Pancreas	82 (50%)
Other*	6 (4%)
**Sample Type**	
Brush Biopsy	2 (1%)
EUS-guided FNB	16 (10%)
US-guided Biopsy	79 (48%)
Surgical Resection	66 (41%)

Supplemental Table 1. Demographics for all patients in the study. Summary of clinical information for patients included in the study for generating PDOs. *Includes retroperitoneal lymph node, soft tissue in the abdomen, next to gastrojejunal anastomosis, peritoneal lesion, abdominal muscle, and lung.

Organoid	Diagnosis	Age	Sex	Race	Initiation Date	Sample Type	Sample Location	Grew	Survived 1st Passage	Survived ≥ 3 Passages	Initiation Media	Reason For Failure
HO1	CCA	65	Male	White	8/18/2017	Brush	Liver	Y	N	NA	PaTOM	Bacterial Contamination
HO2	CCA	71	Male	White	8/22/2017	Brush	Liver	Y	N	NA	PaTOM	Bacterial Contamination
HO3	PDAC	66	Male	White	9/5/2017	FNA	Pancrease	Y	Y	N	PaTOM	Fibroblast Contamination
HO5	PDAC	50	Female	White	9/26/2017	Core Biopsy	Liver	Y	Y	N	PaTOM	Poor Proliferation
HO7	PDAC	60	Male	White	10/24/2017	Core Biopsy	Liver	Y	Y	N	PaTOM	Poor Proliferation
HO8	PDAC	36	Female	White	10/26/2017	Core Biopsy	Liver	Y	N	N	PaTOM	Poor Proliferation
HO9	PDAC	72	Female	White	10/27/2017	FNA	Pancreas	Y	Y	N	PaTOM	Fibroblast Contamination
HO10	PDAC	64	Female	White	11/7/2017	FNA	Pancreas	Y	Y	Y	PaTOM	
HO11	PDAC	71	Male	White	11/9/2017	Core Biopsy	Liver	N	NA	NA	PaTOM	Low Cellularity
HO12	CRC	54	Male	White	11/17/2017	Core Biopsy	Liver	Y	Y	Y	PaTOM	
HO13	PDAC	77	Female	White	11/21/2017	Resection	Pancreas	Y	N	NA	PaTOM	Poor Proliferation
HO14	PDAC	62	Male	White	11/22/2017	Resection	Pancreas	Y	Y	N	PaTOM	Poor Proliferation
HO15	PNET	67	Female	White	12/1/2017	Resection	Pancreas	N	NA	NA	PaTOM	Low Cellularity
HO16	PDAC	59	Male	White	12/4/2017	Core Biopsy	Liver	N	NA	NA	PaTOM	Low Cellularity
HO17	CCA	61	Male	White	12/4/2017	Core Biopsy	Liver	Y	Y	Y	PaTOM	
HO18	CCA	56	Male	White	12/7/2017	Core Biopsy	Liver	N	NA	NA	PaTOM	Low Cellularity
HO19	PDAC	74	Male	White	12/20/2017	Resection	Pancreas	N	NA	NA	PaTOM	Fungal Contamination
HO20	CRC	66	Male	White	12/26/2017	Core Biopsy	Retroperitoneal Lymph Node	Y	Y	Y	PaTOM	
HO21	CRC	64	Female	White	12/29/2017	Core Biopsy	Liver	Y	N	NA	PaTOM	Poor Proliferation
HO22	PDAC	64	Female	White	1/2/2018	Core Biopsy	Abdomen Soft Tissue	N	NA	NA	PaTOM	Low Cellularity
HO23	PDAC	65	Female	White	1/3/2018	Resection	Pancreas	N	NA	NA	PaTOM	Fungal Contamination
HO24	CCA	55	Male	White	1/4/2018	Core Biopsy	Liver	N	NA	NA	PaTOM	Low Cellularity
HO25	PDAC	66	Female	Asian	1/9/2018	Core Biopsy	Liver	N	NA	NA	PaTOM	Low Cellularity
HO26	PDAC	70	Female	White	1/12/2018	Resection	Pancreas	N	NA	NA	PaTOM	Low Cellularity
HO27	PDAC	67	Female	Asian	1/17/2018	FNA	Pancreas	Y	Y	N	PaTOM	Fibroblast Contamination
HO28	PDAC	89	Male	White	1/19/2018	FNA	Pancreas	Y	Y	N	PaTOM, WNT	Poor Proliferation
HO29	PNET	51	Male	White	1/22/2018	Resection	Pancreas	Y	Y	Y	PaTOM	
HO30	PDAC	76	Male	White	1/22/2018	Resection	Pancreas	N	NA	NA	PaTOM	Low Cellularity
HO31	PDAC	71	Male	White	1/23/2018	Resection	Pancreas	N	NA	NA	PaTOM	Low Cellularity
HO32	PDAC	76	Female	White	1/25/2018	Resection	Pancreas	N	NA	NA	PaTOM	Fungal Contamination
HO33	PDAC	78	Male	White	1/25/2018	Resection	Pancreas	N	NA	NA	PaTOM	Low Cellularity
HO34	PDAC	78	Male	White	1/30/2018	Resection	Pancreas	N	NA	NA	PaTOM	Low Cellularity
HO35	PDAC	24	Female	White	2/2/2018	Resection	Pancreas	N	NA	NA	PaTOM	Low Cellularity
HO36	PDAC	73	Male	White	2/5/2018	Resection	Pancreas	Y	Y	N	PaTOM	Poor Proliferation
HO37	CCA	73	Female	White	2/12/2018	Core Biopsy	Liver	N	NA	NA	PaTOM	Low Cellularity
HO38	PDAC	60	Male	White	2/13/2018	Resection	Pancreas	N	NA	NA	PaTOM	Fungal Contamination
HO39	PDAC	83	Female	White	2/19/2018	Resection	Pancreas	Y	N	NA	PaTOM	Fungal Contamination
HO40	CCA	58	Male	White	2/21/2018	Core Biopsy	Liver	N	NA	NA	PaTOM, WNT	Low Cellularity
HO41	PDAC	65	Male	White	2/22/2018	Core Biopsy	Adjacent to Gastrojejunal Anastomosis	N	NA	NA	PaTOM, WNT	Low Cellularity
HO42	CCA	66	Male	White	2/23/2018	Core Biopsy	Liver	Y	Y	Y	PaTOM, WNT	
HO43	PDAC	79	Male	White	2/26/2018	Resection	Pancreas	N	NA	NA	PaTOM	Low Cellularity
HO44	PDAC	71	Female	White	3/13/2018	Resection	Pancreas	Y	Y	Y	PaTOM	
HO45	PNET	72	Male	White	3/14/2018	Resection	Pancreas	Y	Y	N	PaTOM	Fibroblast Contamination
HO46	CRC	54	Male	White	3/19/2018	Core Biopsy	Liver	Y	Y	Y	PaTOM	
HO47	PDAC	25	Female	White	3/21/2018	Resection	Pancreas	N	NA	NA	PaTOM, WNT	Low Cellularity
HO48	PNET	67	Male	White	4/3/2018	Resection	Pancreas	Y	Y	N	PaTOM	Fibroblast Contamination
HO49	PDAC	70	Female	White	4/4/2018	FNA	Pancreas	Y	Y	N	PaTOM, WNT	Poor Proliferation
HO50	PDAC	64	Female	White	4/6/2018	Resection	Pancreas	Y	Y	Y	PaTOM	
HO51	PDAC	76	Male	White	4/11/2018	Resection	Pancreas	N	NA	NA	PaTOM	Low Cellularity
HO52	PNET	57	Male	White	4/13/2018	Resection	Pancreas	N	NA	NA	PaTOM	Low Cellularity
HO53	PNET	67	Male	White	4/17/2018	Resection	Pancreas	N	NA	NA	PaTOM	Low Cellularity
HO54	PDAC	62	Male	White	4/19/2018	Resection	Pancreas	N	NA	NA	PaTOM	Low Cellularity
HO55	CCA	62	Male	White	4/26/2018	Core Biopsy	Liver	N	NA	NA	PaTOM	Low Cellularity
HO56	PDAC	69	Female	White	4/26/2018	FNA	Pancreas	Y	N	NA	PaTOM, WNT	Fibroblast Contamination
HO57	CRC	68	Female	White	5/2/2018	Core Biopsy	Liver	Y	Y	N	PaTOM	Bacterial Contamination
HO58	PDAC	63	Female	White	5/2/2018	Resection	Pancreas	N	NA	NA	PaTOM	Low Cellularity
HO63	PDAC	70	Male	White	6/6/2018	Resection	Pancreas	Y	N	NA	PaTOM	Poor Proliferation
HO65	CRC	84	Male	White	6/21/2018	Core Biopsy	Liver	Y	Y	N	PaTOM	Bacterial Contamination
HO66	PDAC	51	Male	White	6/22/2018	Resection	Pancreas	Y	N	NA	PaTOM	Bacterial Contamination
HO67	PDAC	72	Female	White	7/11/2018	FNA	Pancreas	Y	N	NA	PaTOM	Poor Proliferation
HO69	PDAC	68	Female	White	7/13/2018	FNA	Pancreas	Y	Y	N	PaTOM	Fibroblast Contamination
HO70	PNET	82	Male	White	7/16/2018	Resection	Pancreas	N	NA	NA	PaTOM	Low Cellularity
HO71	PDAC	56	Female	White	7/18/2018	Core Biopsy	Liver	N	NA	NA	PaTOM	Low Cellularity
HO72	PNET	61	Male	White	7/24/2018	Resection	Pancreas	Y	Y	N	PaTOM	Poor Proliferation
HO73	PDAC	70	Female	White	7/31/2018	FNA	Liver	N	NA	NA	PaTOM	Low Cellularity
HO77	CCA	62	Male	White	8/6/2018	Core Biopsy	Liver	Y	N	NA	PaTOM	Poor Proliferation
HO79	PDAC	71	Male	White	8/14/2018	Resection	Pancreas	N	NA	NA	PaTOM	Low Cellularity
HO81	CRC	59	Male	White	8/20/2018	Core Biopsy	Liver	N	NA	NA	PaTOM	Low Cellularity
HO83	PDAC	76	Male	White	8/23/2018	Resection	Pancreas	N	NA	NA	PaTOM	Fungal Contamination
HO84	PDAC	64	Male	White	8/24/2018	Resection	Pancreas	N	NA	NA	PaTOM	Fungal Contamination
HO86	PDAC	63	Male	White	8/29/2018	FNA	Pancreas	N	NA	NA	PaTOM	Low Cellularity
HO87	PDAC	64	Female	White	8/29/2018	Resection	Pancreas	Y	N	NA	PaTOM	Fungal Contamination
HO90	CRC	37	Male	White	9/19/2018	Core Biopsy	Liver	Y	Y	Y	PaTOM	
HO92	CCA	64	Female	White	9/24/2018	Core Biopsy	Liver	N	NA	NA	PaTOM	Low Cellularity
HO93	PDAC	76	Female	White	9/25/2018	Resection	Pancreas	N	N	NA	PaTOM	Low Cellularity
HO94	PDAC	70	Male	White	9/28/2018	FNA	Pancreas	Y	N	NA	PaTOM	Poor Proliferation
HO95	PDAC	79	Male	White	9/28/2018	Resection	Pancreas	N	NA	NA	PaTOM	Low Cellularity
HO96	PDAC	65	Female	White	10/2/2018	Resection	Pancreas	N	NA	NA	PaTOM	Low Cellularity
HO97	PDAC	65	Female	White	10/9/2018	Resection	Pancreas	Y	Y	Y	PaTOM	
HO98	CRC	66	Male	White	11/15/2018	Core Biopsy	Liver	Y	Y	Y	PaTOM	
HO99	PDAC	47	Male	White	11/15/2018	Core Biopsy	Liver	Y	Y	N	PaTOM, WNT	Poor Proliferation
HO100	PDAC	67	Female	White	11/20/2018	Resection	Pancreas	Y	Y	N	PaTOM, WNT	Poor Proliferation
HO101	CRC	56	Female	White	12/7/2018	Core Biopsy	Peritoneal Lesion	N	NA	NA	PaTOM	Low Cellularity
HO102	PDAC	79	Male	White	12/11/2018	Resection	Pancreas	N	NA	NA	PaTOM	Low Cellularity
HO103	PDAC	70	Male	White	12/14/2018	FNA	Pancreas	Y	Y	N	PaTOM, WNT	Poor Proliferation

HO104	PDAC	53	Male	White	12/18/2018	Core Biopsy	Liver	N	NA	NA	PaTOM	Low Cellularity
HO105	CCA	63	Male	White	12/21/2018	Core Biopsy	Liver	N	NA	NA	PaTOM	Low Cellularity
HO106	Small Bowel	68	Female	White	12/28/2018	Core Biopsy	Liver	Y	Y	Y	PaTOM	
HO107	PDAC	77	Female	White	1/21/2019	Core Biopsy	Liver	Y	Y	Y	PaTOM, WNT	
HO108	PNET	65	Male	White	1/23/2019	Resection	Pancreas	N	NA	NA	PaTOM	Poor Proliferation
HO110	PDAC	70	Female	White	2/8/2019	Core Biopsy	Liver	Y	N	NA	PaTOM	Low Cellularity
HO111	PDAC	78	Female	White	3/4/2019	Resection	Pancreas	N	NA	NA	PaTOM	Low Cellularity
HO112	PDAC	70	Female	White	3/5/2019	Resection	Pancreas	N	NA	NA	PaTOM	Fungal Contamination
HO113	PDAC	58	Male	White	3/6/2019	Resection	Pancreas	Y	Y	Y	PaTOM, WNT	
HO114	PDAC	87	Female	White	3/8/2019	Resection	Pancreas	N	NA	NA	PaTOM	Fungal Contamination
HO115	CCA	61	Female	White	4/1/2019	Core biopsy	Liver	N	NA	NA	PaTOM	Low Cellularity
HO116	PNET	67	Male	White	4/8/2019	Resection	Pancreas	Y	Y	Y	PaTOM, WNT	
HO117	CCA	58	Female	White	4/18/2019	Core Biopsy	Liver	N	NA	NA	PaTOM	Low Cellularity
HO118	PNET	71	Male	White	4/22/2019	Resection	Pancreas	N	NA	NA	PaTOM	Fungal Contamination
HO119	PDAC	75	Male	Asian	4/22/2019	Resection	Pancreas	N	NA	NA	PaTOM	Fungal Contamination
HO120	PDAC	59	Male	White	4/25/2019	FNA	Pancreas	Y	N	NA	PaTOM, WNT	Low Cellularity
HO121	PDAC	71	Female	White	4/30/2019	Resection	Pancreas	N	NA	NA	PaTOM, WNT	Poor Proliferation
HO122	PDAC	55	Female	White	5/1/2019	Resection	Pancreas	N	NA	NA	PaTOM, WNT	Low Cellularity
HO123	CRC	64	Male	White	5/3/2019	Core Biopsy	Liver	Y	Y	Y	PaTOM	
HO124	PDAC	87	Female	White	5/8/2019	Resection	Pancreas	Y	N	NA	WNT	Poor Proliferation
HO126	CRC	55	Female	White	5/20/2019	Core Biopsy	Liver	N	NA	NA	PaTOM	Poor Proliferation
HO127	CRC	44	Female	White	5/22/2019	Core Biopsy	Liver	Y	Y	Y	PaTOM	
HO128	CCA	76	Male	White	5/30/2019	Core Biopsy	Liver	Y	N	NA	PaTOM, WNT	Low Cellularity
HO129	PNET	73	Female	White	6/5/2019	Resection	Pancreas	Y	Y	Y	WNT	
HO130	ACC	58	Male	White	6/10/2019	Core Biopsy	Liver	Y	Y	Y	WNT	
HO131	CCA	50	Female	White	6/17/2019	Core Biopsy	Liver	Y	Y	Y	WNT	
HO132	CCA	59	Male	White	6/17/2019	Core Biopsy	Liver	Y	N	NA	WNT	Poor Proliferation
HO133	PDAC	67	Male	White	6/18/2019	Resection	Pancreas	Y	Y	Y	WNT	
HO134	PNET	37	Female	White	6/24/2019	Resection	Pancreas	Y	Y	Y	WNT	
HO135	PDAC	72	Female	White	6/24/2019	FNA	Pancreas	Y	Y	Y	PaTOM, WNT	
HO136	CRC	66	Male	White	7/5/2019	Core Biopsy	Liver	N	NA	NA	WNT	Poor Proliferation
HO137	ACC	47	Female	White	7/11/2019	Core Biopsy	Liver	Y	Y	Y	WNT	
HO138	PDAC	62	Female	White	7/18/2019	Resection	Pancreas	N	NA	NA	WNT	Low Cellularity
HO139	CCA	42	Male	White	7/18/2019	Core Biopsy	Liver	N	NA	NA	WNT	Low Cellularity
HO140	CRC	29	Male	Asian	7/23/2019	Core Biopsy	Liver	Y	Y	Y	PaTOM	
HO141	PDAC	60	Male	White	7/26/2019	Core Biopsy	Pancreas	N	NA	NA	WNT	Low Cellularity
HO142	PDAC	66	Female	White	7/26/2019	Resection	Pancreas	Y	Y	Y	WNT	
HO143	PDAC	66	Female	White	7/29/2019	Core Biopsy	Liver	Y	N	NA	WNT	Poor Proliferation
HO144	CCA	45	Male	White	7/30/2019	Core Biopsy	Liver	N	NA	NA	WNT	Low Cellularity
HO145	PDAC	65	Female	White	7/31/2019	Core Biopsy	Liver	Y	Y	Y	WNT	
HO146	PDAC	41	Male	White	8/1/2019	Core Biopsy	Liver	N	NA	NA	WNT	Low Cellularity
HO147	CCA	78	Male	White	8/7/2019	Core Biopsy	Abdominal Muscle	N	NA	NA	WNT	Low Cellularity
HO148	CCA	77	Male	White	8/7/2019	Core Biopsy	Liver	N	NA	NA	WNT	Low Cellularity
HO149	PDAC	64	Male	White	8/9/2019	Resection	Pancreas	N	NA	NA	WNT	Low Cellularity
HO150	PDAC	78	Male	White	8/13/2019	Resection	Pancreas	N	NA	NA	WNT	Low Cellularity
HO151	PDAC	83	Male	White	8/15/2019	FNA	Pancreas	N	NA	NA	WNT	Low Cellularity
HO152	PDAC	53	Male	White	8/20/2019	Resection	Pancreas	N	NA	NA	WNT	Low Cellularity
HO153	PDAC	74	Male	White	8/27/2019	Resection	Pancreas	Y	Y	Y	WNT	
HO154	PDAC	71	Female	White	8/27/2019	Resection	Pancreas	Y	Y	Y	WNT	
HO155	PDAC	57	Female	White	8/30/2019	Resection	Pancreas	N	NA	NA	WNT	Low Cellularity
HO156	PDAC	67	Female	White	9/4/2019	Resection	Pancreas	N	NA	NA	WNT	Low Cellularity
HO157	PDAC	71	Male	White	9/6/2019	Resection	Pancreas	N	NA	NA	WNT	Low Cellularity
HO165	PDAC	66	Male	White	10/8/2019	Core Biopsy	Liver	Y	Y	Y	WNT	
HO166	CCA	61	Male	White	10/28/2019	Core Biopsy	Liver	N	NA	NA	WNT	Low Cellularity
HO167	CCA	73	Female	White	10/28/2019	Core Biopsy	Liver	N	NA	NA	WNT	Low Cellularity
HO169	CCA	50	Female	White	12/5/2019	Core Biopsy	Liver	Y	Y	N	WNT	Fibroblast Contamination
HO170	CCA	61	Male	White	12/5/2019	Core Biopsy	Liver	Y	Y	Y	WNT	
HO171	CCA	69	Female	White	12/16/2019	Core Biopsy	Liver	Y	Y	Y	WNT	
HO172	PDAC	70	Female	White	12/16/2019	Resection	Pancreas	Y	Y	N	WNT	Fibroblast Contamination
HO173	PDAC	66	Male	White	12/26/2019	Core Biopsy	Liver	N	NA	NA	WNT	Low Cellularity
HO181	CCA	72	Male	White	1/8/2020	Core Biopsy	Liver	Y	Y	N	WNT	Poor Proliferation
HO182	CCA	71	Male	White	1/15/2020	Core Biopsy	Liver	N	NA	NA	WNT	Low Cellularity
HO183	CCA	73	Female	White	1/15/2020	Core Biopsy	Liver	N	NA	NA	WNT	Low Cellularity
HO184	CCA	58	Female	White	1/31/2020	Core Biopsy	Liver	Y	Y	N	WNT	Bacterial Contamination
HO185	CCA	55	Female	White	2/6/2020	Core Biopsy	Liver	Y	Y	N	WNT	Poor Proliferation
HO186	CCA	72	Male	White	2/12/2020	Core Biopsy	Liver	Y	Y	N	WNT	Bacterial Contamination
HO187	CCA	61	Female	White	2/13/2020	Core Biopsy	Liver	Y	Y	N	WNT	Fibroblast Contamination
HO190	PDAC	67	Female	White	2/27/2020	Core Biopsy	Liver	Y	Y	Y	WNT	
HO191	CCA	79	Male	White	2/27/2020	Core Biopsy	Liver	Y	N	N	WNT	Fibroblast Contamination
HO192	PDAC	64	Female	White	3/3/2020	Core Biopsy	Liver	N	NA	NA	WNT	Low Cellularity
HO193	CCA	81	Female	White	3/11/2020	Core Biopsy	Liver	Y	Y	Y	WNT	
HO213	CCA	77	Male	White	7/23/2020	Core biopsy	Liver	Y	N	NA	WNT	Poor Proliferation
HO214	CCA	49	Female	White	8/6/2020	Core Biopsy	Liver	N	N	NA	WNT	Poor Proliferation
HO215	CCA	56	Female	White	8/20/2020	Core Biopsy	Liver	N	N	N	WNT	Poor Proliferation
HO216	CCA	81	Male	White	9/3/2020	Core Biopsy	Liver	N	N	N	WNT	Poor Proliferation
HO220	CCA	71	Female	African American	10/21/2020	Core Biopsy	Liver	Y	N	N	WNT	Poor Proliferation
HO230	CCA	71	Male	White	11/10/2020	Core Biopsy	Liver	Y	Y	N	WNT	Poor Proliferation
HO232	PDAC	67	Male	White	1/21/2021	Core Biopsy	Pancreas	Y	Y	Y	WNT	

Supplemental Table 2. List of all donor patient PDO information for the study. Information includes lab organoid ID, diagnosis, age, sex, race, PDO initiation date, sample type, sample location, PDO initiation success summary, culture media, and reason for PDO failure (if applicable). Demographic options and classifications were made by the investigators.

PDO ID	Source	Diagnosis	Tumor Stage	Sample Type	Media	IF	Donor Tumor Sequence	PDO DNA-Seq	RNA-Seq	CGH (CNV)
**HO12**	Mayo IRB	CRC	4	Biopsy (liver)	PaTOM	CK19, CDH1	Tempus	Onco-Seq Panel	NA	P1, P5
**HO17***	Mayo IRB	CCA	4	Biopsy (liver)	PaTOM	CK19, SOX9	Tempus	Onco-Seq Panel	NA	P8, P30
**HO20***	Mayo IRB	CRC	4	Biopsy (lymph node)	PaTOM	CK19, CDH1, MUC2	Tempus	Onco-Seq Panel	NA	P3, P10
**HO29**	Mayo IRB	PNET	2	Resection	PaTOM	Syn, Chrom	NA	Onco-Seq Panel	NA	NA
**HO42***	Mayo IRB	CCA	4	Biopsy (liver)	PaTOM	NA	NA	QIAseq Panel	NA	NA
**HO44**	Mayo IRB	PDAC	2	Resection	PaTOM<br>WNT	CK19, CDH1, SOX9	NA	Onco-Seq Panel	WNT P15,P16,P17<br>PaTOM P16,P17,P25	NA
**HO46***	Mayo IRB	CRC	4	Biopsy (liver)	PaTOM	NA	Tempus	QIAseq Panel	NA	NA
**HO90***	Mayo IRB	CRC	4	Biopsy (liver)	PaTOM	NA	Tempus	Onco-Seq Panel	NA	NA
**HO98***	Mayo IRB	CRC	4	Biopsy (liver)	PaTOM	NA	Tempus	Onco-Seq Panel	NA	P1, P19
**HO106***	Mayo IRB	Small Bowel	4	Biopsy (liver)	PaTOM	NA	Tempus	Onco-Seq Panel	NA	NA
**HO107***	Mayo IRB	PDAC	4	Biopsy (liver)	PaTOM<br>WNT	NA	Tempus	QIAseq Panel	WNT P12,P13<br>PaTOM P10,P17	NA
**HO123***	Mayo IRB	CRC	4	Biopsy (liver)	PaTOM	NA	Tempus	QIAseq Panel	NA	NA
**HO127***	Mayo IRB	CRC	4	Biopsy (liver)	PaTOM	NA	Tempus	QIAseq Panel	NA	NA
**HO133**	Mayo IRB	PDAC	2	Resection	WNT	NA	NA	QIAseq Panel	NA	NA
**HO140***	Mayo IRB	CRC	4	Biopsy (liver)	PaTOM	NA	Tempus	QIAseq Panel	NA	NA
**HO145**	Mayo IRB	PDAC	4	Biopsy (liver)	WNT	NA	NA	QIAseq Panel	NA	NA
**HO153**	Mayo IRB	PDAC	2	Resection	WNT	NA	NA	QIAseq Panel	NA	NA
**HO159**	France IRB	PDAC	1/2	PDX Tumor	WNT	NA	NA	QIAseq Panel	NA	NA
**HO160**	France IRB	PDAC	1/2	PDX Tumor	WNT	NA	NA	QIAseq Panel	NA	NA
**HO161**	France IRB	PDAC	1/2	PDX Tumor	WNT	NA	NA	QIAseq Panel	NA	NA
**HO162**	Mayo IRB	PDAC	4	PDX Tumor	PaTOM	NA	NA	NA	NA	NA
**HO163**	Mayo IRB	PDAC	4	PDX Tumor	PaTOM<br>WNT	NA	NA	NA	WNT P6,P7,P8,P12<br>PaTOM P6,P7,P8,P12	NA
**HO165**	Mayo IRB	PDAC	4	Biopsy (liver)	WNT	NA	NA	QIAseq Panel	NA	NA
**HO219**	Mayo IRB	PDAC	2	PDX Tumor	PaTOM<br>WNT	NA	NA	NA	WNT P18,P19,P20<br>PaTOM P18,P19,P20	NA
**HO222**	Mayo IRB	PDAC	2	PDX Tumor	PaTOM	NA	NA	NA	NA	NA
**HO227**	Mayo IRB	PDAC	2	PDX Tumor	PaTOM	NA	NA	NA	NA	NA
**HO228**	Mayo IRB	PDAC	2	PDX Tumor	PaTOM<br>WNT	NA	NA	NA	WNT P21,P22,P23<br>PaTOM P21,P22,P23,P24	NA
**HO231**	Mayo IRB	PDAC	2	PDX Tumor	PaTOM	NA	NA	NA	NA	NA

Supplemental Table 3. Information for all PDOs in the study. Information includes PDO lab ID, the institution from which the tissue for the PDO was obtained (source), diagnosis of the donor patient, stage of the tumor at the time of specimen collection, sample type, media in which the PDO was grown, markers detected using IF in PDOs, specimens with donor tumor sequence available (for mutation comparison to corresponding PDO) from the Tempus Targeted sequencing panel, and PDOs in which we performed DNA-seq, RNA seq- and comparative genomic hybridization (CGH) for CNV analysis. PDOs with an asterisk next to their number were utilized for our clinical correlation studies in Figure 5. Syn = Synaptophysin, Chrom = Chromogranin, P=Passage number, NA = Not Available (not performed).

Supplemental Table 4 (Excel File). GSEA Hallmark Gene sets enriched in HO107 and HO44 in PaTOM.

Supplemental Table 5 (Excel File). GSEA Hallmark and KEGG Gene sets enriched in WNT and PaTOM.

PDO ID	Patient Diagnosis	Lines Pre-Sample	Patient Treatment History Pre-Sample	Biopsy Date	Patient Treatment History Post-Sample	CT Scans Dates	Patient Treatment Evaluated	Patient Response	Organoid Treatment	Organoid Response	Overall Correlation	IC_{50} References
HO17	CCA	3	(**1**) FOLFIRINOX w/ SD, (**2**) Gem+Nab+Paclitaxel w/ PD, (**3**) Pembro w/ PD	12/4/2017	(**1**) **(11/2017 to 1/15/2018) Gemcitabine + Erlotinib w/ PD.**	11/20/17, 1/14/18	Gemcitabine + Erlotonib	PD	Gemcitabine	Resistant (IC_{50} = 15.6 nM)	MATCH	IC_{50} greater than 10 nM associated with Resistance [1]. IC_{50} less than 5 nM associated with Sensitivity [2].
									Erlotonib	Resistant (IC_{50} > 1 µM)		IC_{50} greater than 1 µM is associated with Resistance [3].
HO42	CCA	2	(**1**) Cisplatin+Gemcitabine w/ PD, (**2**) FOLFOX x4 w/ PD	2/23/2018	(**1**) **(2/22/2018 to 3/2018) Clinical trial with TAS-102, complicated by anemia and biliary obstruction w/ PD.**	2/20/18, 3/15/18 (MRI)	TAS-102	PD	TAS-102	Sensitive (IC_{50} = 303 nM)	MISMATCH	IC_{50} greater than 1 µM associated with Resistance [4].
HO107	PDAC	4	(**1**) Neoadjuvant Gemcitabine/Abraxane w/ PR, (**2**) FOLFIRINOX x 1 dose, then 5-FU/liposomal irinotecan (PD). (**3**) Gemcitabine / Abraxane / Cisplatin w/ PD, (**4**) **(10/2018-11/2018) FOLFOX w/ PD**	1/21/2019	None	10/25/2018, 11/26/2018	FOLFOX	PD	5-FU	Resistant	MATCH	NA
									Oxaliplatin	Intermediate		
HO20	CRC	3	(**1**) FOLFOX x12, (**2**) FOLFIRI+Bev w/ PD, (**3**) FOLFIRI+Panitumumab w/ PD	12/26/2017	(**1**) (1/2018 to 7/2018) BACCI trial (Atezolizumab, Capecitabine, and Avastin versus Placebo plus Capecitabine and Avastin; blinded) w/ PD. (**2**) (8/2018) Clinical Trial MK-4280 trial with anti-LAG3 and Pembrolizumab. Discontinued after 1 dose. (**3**) **(9/2018 to 2/2019) Vemurafenib + Panitumumab w/ PR in adrenal metastases.**	8/24/2018, 11/12/2018	Vemurafenib + Panitumumab	PR	Vemurafenib	Sensitive	MATCH	NA
									Cetuximab	Resistant		
HO46	CRC	4	(**1**) Neoadjuvant FOLFOX+Bev w/ PR (**2**) Adjuvant Capecitabine w/ PD (**3**) FOLFIRI+Bev w/ PR, (4) 5-FU+Bev w/ SD (**4**) FOLFIRI+Bev w/ PD	3/19/2018	(**1**) **(4/18/2018 to 6/20/2018) Clinical trial with avelumab + eFT508 w/ PD**	4/17/18, 6/20/18	eFT508 + avelumab	PD	eft508	Resistant	MATCH	NA
HO98	CRC	4	(**1**) FOLFOX+Bev w/ PR, (**2**) FOLFIRI+Cetuximab w/ PD, (**3**) TAS-102 w/ PD (**4**) Regorafenib w/ PD	11/15/2018	(**1**) **(3/4/2019 to 4/29/2019) Clinical trial with CBT-101-01 Bozitinib w/ PD**	2/12/19, 4/29/19	Bozitinib	PD	Bozitinib	Resistant	MATCH	NA
HO90	CRC	0	None	9/19/2018	(**1**) **(10/4/2018 to 11/17/2018) Regorafenib w/ SD.**	10/6/2018, 11/26/2018	Regorafenib	SD	Regorafenib	Intermediate	MATCH	NA
HO123	CRC	4	(**1**) FOLFOX w/ PD, (**2**) FOLFIRI+Bev w/ PD, (**3**) TAS-102 w/ PD (**4**) **(11/2018 to 1/2019) Regorafenib w/ PD**	5/3/2019	(**1**) (5/6/2019) Clinical trial with KO-ERK-001	10/18/18, 2/1/19	Regorafenib	PD	Regorafenib	Resistant	MATCH	NA
HO127	CRC	1	(**1**) **(1/30/19 to 5/17/19) FOLFOX+Bev w/ PD**	5/22/2019	(**1**) (6/3/2019 to 9/25/2019) Clinical trial BMS-813160 + FOLFIRI w/ PD	1/17/19, 4/8/19	FOLFOX + Bevacizumab	PD	5-FU	Intermediate	MATCH	NA
									Oxaliplatin	Resistant		
HO140	CRC	0	None	7/23/2019	(**1**) **(8/1/2019 to 12/2019) CAPOX + Panitumumab w/ PR**	7/16/19, 10/3/19	CAPOX + Panitumumab	PR	5-FU	Resistant	MISMATCH	NA
									Oxaliplatin	Resistant		
									Cetuximab	Intermediate		
HO106	Small Bowel Cancer	3	(**1**) FOLFIRI w/ PD, (**2**) FOLFOX+Bev w/ PD, (**3**) FOLFOX+Panitumumab w/ PD	12/28/2018	(**1**) **(1/31/2019 to 2/14/2019) Paclitaxel w/ PD**	12/19/18, 2/28/19	Paclitaxel	PD	Paclitaxel	Intermediate	MATCH	NA

Supplemental Table 6. Extended data for donor patients and corresponding PDOs used for the clinical correlation study. Relevant clinical information for patients included in the correlative study and their corresponding PDO, including patient treatment history and response, PDO treatment and response, and correlation. The treatment listed in **bold text** is the treatment evaluated for clinical correlation.

Supplemental Method References

1. Varamo, C., et al., *Establishment and Characterization of a New Intrahepatic Cholangiocarcinoma Cell Line Resistant to Gemcitabine.* Cancers (Basel), 2019. **11**(4).
2. Tiriac, H., et al., *Organoid Profiling Identifies Common Responders to Chemotherapy in Pancreatic Cancer.* Cancer Discov, 2018. **8**(9): p. 1112-1129.
3. Saito, Y., et al., *Establishment of Patient-Derived Organoids and Drug Screening for Biliary Tract Carcinoma.* Cell Rep, 2019. **27**(4): p. 1265-1276 e4.
4. Vlachogiannis, G., et al., *Patient-derived organoids model treatment response of metastatic gastrointestinal cancers.* Science, 2018. **359**(6378): p. 920-926.
